# A Study of the Synergistic Effects of Essential Oils from *Origanum compactum* and *Origanum elongatum* with Commercial Antibiotics against Highly Prioritized Multidrug-Resistant Bacteria for the World Health Organization

**DOI:** 10.3390/metabo14040210

**Published:** 2024-04-07

**Authors:** Aziz Drioiche, Soukayna Baammi, Khalid Zibouh, Omkulthom Al Kamaly, Anwar M. Alnakhli, Firdaous Remok, Soukaina Saidi, Rachid Amaiach, Fadoua El Makhoukhi, Abdelhakim Elomri, Touriya Zair

**Affiliations:** 1 Research Team of Chemistry of Bioactive Molecules and the Environment, Laboratory of Innovative Materials and Biotechnology of Natural Resources, Faculty of Sciences, Moulay Ismail University, B.P. 11201, Zitoune, Meknes 50070, Morocco; k.zibouh@edu.umi.ac.ma (K.Z.); f.remok@edu.umi.ac.ma (F.R.); soukaina.saidi@usms.ma (S.S.); elmakhoukhi@cnrst.ma (F.E.M.); 2 Medical Microbiology Laboratory, Mohamed V. Hospital, Meknes 50000, Morocco; 3 Bioinformatics Laboratory, College of Computing, Mohammed VI Polytechnic University, Ben Guerir 43150, Morocco; soukayna.baammi@um6p.ma; 4 Department of Pharmaceutical Sciences, College of Pharmacy, Princess Nourah bint Abdulrahman University, P.O. Box 84428, Riyadh 11671, Saudi Arabia; omalkmali@pnu.edu.sa (O.A.K.); amalnklee@pnu.edu.sa (A.M.A.); 5 Laboratory of Materials, Processes, Catalysis and Environment, School of Technology, University Sidi Mohamed Ben Abdellah, Fez 30000, Morocco; rachid.amaiach@usmba.ac.ma; 6 UNIROUEN, INSA Rouen, CNRS, COBRA (UMR 6014), Normandie University, 76000 Rouen, France; hakim.elomri@univ-rouen.fr

**Keywords:** *Origanum compactum*, *Origanum elongatum*, thymol, (E)-caryophyllene, carvacrol, antimicrobial activity, drug-resistant bacteria

## Abstract

The irrational use of antibiotics has favored the emergence of resistant bacteria, posing a serious threat to global health. To counteract antibiotic resistance, this research seeks to identify novel antimicrobials derived from essential oils that operate through several mechanisms. It aims to evaluate the quality and composition of essential oils from *Origanum compactum* and *Origanum elongatum*; test their antimicrobial activity against various strains; explore their synergies with commercial antibiotics; predict the efficacy, toxicity, and stability of compounds; and understand their molecular interactions through docking and dynamic simulations. The essential oils were extracted via hydrodistillation from the flowering tops of oregano in the Middle Atlas Mountains in Morocco. Gas chromatography combined with mass spectrometry (GC-MS) was used to examine their composition. Nine common antibiotics were chosen and tested alone or in combination with essential oils to discover synergistic effects against clinically important and resistant bacterial strains. A comprehensive in silico study was conducted, involving molecular docking and molecular dynamics simulations (MD). *O. elongatum* oil includes borneol (8.58%), p-cymene (42.56%), thymol (28.43%), and carvacrol (30.89%), whereas *O. compactum* oil is mostly composed of γ-terpinene (22.89%), p-cymene (15.84%), thymol (10.21%), and (E)-caryophyllene (3.63%). With *O. compactum* proving to be the most potent, these essential oils showed antibacterial action against both Gram-positive and Gram-negative bacteria. Certain antibiotics, including ciprofloxacin, ceftriaxone, amoxicillin, and ampicillin, have been shown to elicit synergistic effects. To fight resistant bacteria, the essential oils of *O. compactum* and *O. elongatum*, particularly those high in thymol and (E)-caryophyllene, seem promising when combined with antibiotics. These synergistic effects could result from their ability to target the same bacterial proteins or facilitate access to target sites, as suggested by molecular docking simulations. Molecular dynamics simulations validated the stability of the examined protein–ligand complexes, emphasizing the propensity of substances like thymol and (E)-caryophyllene for particular target proteins, opening the door to potentially effective new therapeutic approaches against pathogens resistant to multiple drugs.

## 1. Introduction

The development of antibiotics has revolutionized public health and prevented millions of deaths. However, the excessive and irrational use of these drugs has led to their dispersion in the environment, promoting the emergence of antibiotic-resistant bacteria [1]. These multidrug-resistant bacteria are one of the top threats to global food security, development, and health, according to a report by the WHO [2].

The use of antibiotics has been on the rise in recent decades. Global antibiotic consumption increased by 46% between 2000 and 2010 [3]. This large quantity of antibiotics ends up in wastewater, and even if it reaches treatment plants, they fail to eliminate these residues, releasing them into water bodies [4]. Antibiotic residues in high amounts have been found in effluents; some of these residues are applied as fertilizers to the soil.

This widespread dissemination of antibiotic residues in the environment promotes selective pressure and the spread of resistance genes. Antibiotic resistance can result from mutations that modify the molecular targets of bacterial antibiotics [5]. Multidrug-resistant pathogen infections are becoming more difficult to treat, which highlights the need for novel antimicrobial compounds with distinct mechanisms of action that can reduce resistance and, if possible, have fewer negative effects on the health of humans, animals, and the environment following the “One Health” philosophy [6].

To battle strains that are resistant to several drugs, the WHO has created an action plan that centers on the creation of novel antimicrobial products. The search for natural compounds that may be effective as novel antibacterial agents has therefore been quite active.

Numerous natural plant-based medications, such as essential oils (EOs), have been investigated for the treatment and prevention of multidrug-resistant micro-organisms [7,8,9]. Unfortunately, natural products generally have weaker antibiotic activity than common antibiotics; therefore, it is challenging for them to effectively replace current antibiotics in clinical practice. Nonetheless, it has been shown that a small number of plant-derived antimicrobial compounds may work in concert to boost antibiotic action [10,11]. By lowering the minimum inhibitory concentration (MIC) of both the antibiotic and the natural product, the synergistic interaction of natural compounds with commercial antibiotics can make the combination as effective as the antibiotic alone while preserving the use of commercial antibiotics (ATBs) [12,13].

Since combinations with synergistic effects can lower the likelihood of bacterial resistance emergence while maintaining effective pharmacological outcomes, using lower concentrations of both agents presents significant opportunities for exploring alternative solutions for the treatment of infectious diseases [14]. Furthermore, as opposed to the side effects from large doses of synthetic medications, this may result in less toxicity from antibiotics [15].

Polyphenols, alkaloids, carotenoids, terpenes, terpenoids, and sulfur compounds are among the phytochemical substances showing promising antimicrobial activity in vitro [16]. Global demands for antibiotics are increasing, but antibiotic research programs are significantly inadequate; for example, no new class of Gram-negative antibiotics has been introduced in over 50 years [17]. Therefore, medicinal plants can represent a rapid and safe source of innovation for new antimicrobial agents.

The *Lamiaceae* family of plants is one of the most significant for yielding essential oils as it has antibacterial and antioxidant characteristics. The majority of aromatic plants that are high in essential oils are found in the Mediterranean region, where the extraction of oil is a profitable industry that promotes both ecological and economic growth. Species from the genus *Origanum* are among these aromatic and therapeutic plants that are often used as spices due to their abundance of essential oils. Additionally, they display a range of biological activities, the potential of which has been demonstrated by numerous scientific investigations [18,19,20]. The *Lamiaceae* family includes the genus *Origanum*, which is known as “Oregano” in English. In total, 38 species, including 6 subspecies and 17 hybrids, are found in the Mediterranean, Irano-Turanian, and Euro-Siberian regions. Moreover, it is divided into 10 parts [20]. Its medicinal use goes back thousands of years because of its many culinary and therapeutic uses. The leaves were used as an antiseptic and for healing skin blemishes. The ancient Greek and Roman empires also employed them to treat various ailments, like indigestion, diarrhea, and asthma [21]. A common treatment for colds and stomachaches in Greece is still an oregano infusion [22]. Locally known as “Zaatar” or “Zwi” in Berber, oregano species are highly prized in Morocco. While the Zwi aqueous infusion is used to treat dysentery, colitis, bronchopulmonary diseases, gastric acidity, and gastrointestinal ailments, this plant is traditionally used to cure liver disorders in particular places, such as the Middle Atlas (Jbel-Bouiblane) [23]. Additionally, several ethnic groups have been using Origanum species in traditional medicine since antiquity to cure and relieve a wide range of ailments. In addition to treating other illnesses, they have antibacterial, anti-inflammatory, antioxidant, anticancer, antifungal, antiviral, and antileishmanial properties [24,25,26].

Finding combinations of these EOs with various classes of commercial antibiotics to find those that have synergistic effects and may allow the use of lower antibiotic doses is the aim of this study. *Origanum compactum* and *Origanum elongatum* EOs were harvested in different regions of Morocco. The chemical composition of the EOs was identified. We investigated the synergistic combinations by comparing the minimum inhibitory concentrations of the EOs under investigation with nine commonly used antibiotics, both in isolation and in combination, against microbial strains that cause a variety of human diseases. Following the WHO’s list of priority pathogens, the types of bacteria were selected based on their potential severity and capacity for resistance development, as well as their clinical significance as they cause some of the most prevalent diseases in existence today [27]. Lastly, to gain a deeper comprehension of the fundamental mechanisms behind our experimental endeavors, we will employ drug similarity prediction, molecular dynamics, pharmacokinetics (ADME-Tox), and molecular docking simulations.

## 2. Materials and Methods

### 2.1. Plant Material

**Figure 1:**
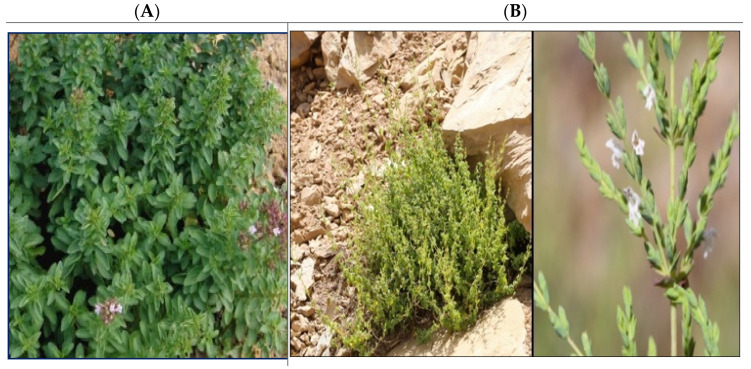
Morphological aspect of the studied oregano species; (**A**) *O. compactum* and (**B**) *O. elongatum*.

In July 2022, the flowering tops of *Origanum compactum* and *Origanum elongatum* were collected in full bloom in the Moroccan Middle Atlas highlands of Bouyablane and Khenifra. At the Scientific Institute of Rabat’s botany lab, species identification was performed. The plants were subsequently identified and verified at the Scientific Institute of Rabat’s Department of Botany after being dried in the shade for around ten days. Table 1 and Table 2, together with Figure 1, offer comprehensive details on each species.

### 2.2. Extraction of Essential Oils

Clevenger-type apparatus equipment was used to hydrodistillate the essential oils for three hours to extract them. For every 100 g of dry matter, the yield of essential oil was calculated in milliliters. The extracted essential oil was kept out of direct sunlight at 4 °C.

### 2.3. Gas Chromatography Coupled with Mass Spectrometry Analysis of Essential Oils

A Thermo Electron mass spectrometer (Thermo Electron: Trace GC Ultra; Polaris Q MS) and gas chromatograph (Trace GC Ultra, Milan, Italy) were used for the chromatographic examination of the examined essential oil sample. By employing an electron collision with an energy level of 70 eV, fragmentation was accomplished. A flame ionization detector (FID) and a DB-5-type column were fitted to the chromatograph. The temperature of the column was set to rise for five minutes, from 50 to 200 °C at a pace of 4 °C per minute. The carrier gas was nitrogen, which was injected in split mode at a rate of 1 mL/min (leak ratio: 1/70). The Kovats indices (KI) of compounds were calculated and compared to those of standard products listed in the Kovats [28], Adams [29], and Hübschmann [30], databases to determine the chemical composition of the essential oil. Each molecule was identified by comparing its retention time to genuine standards that were known to be kept in the laboratory of the authors. Their stated KI and MS data were also verified against the published literature and standards included in the WILEY and NIST 14 standard mass spectrum databases. This was carried out using the Kovats index. To compare the retention periods of any products to the retention times of linear alkanes with an equivalent carbon content, Kovats indices were employed. By simultaneously infusing a traditional C_7_–C_40_ alkane combination under the same operating circumstances, they were established.

### 2.4. Antimicrobial Compounds

The nine antibiotics that were examined were chosen because they represent various modes of action and are among the most widely used antibiotics on the market today. The suppliers of all antibiotics were Sigma Aldrich in Darmstadt, Germany, and Acofarma in Barcelona, Spain. Table 3 presents a comprehensive overview of every chemical.

### 2.5. Micro-Organisms

Twenty-nine bacterial strains (Gram-positive cocci and Gram-negative bacilli) and eight fungal strains (yeasts and molds) were subjected to the antibacterial activity (Table 4). These particular microbes are highly pathogenic and are well known for their invasiveness, potency, and toxicity to humans. They are often seen in Morocco in a variety of infections that present both clinical and treatment challenges. These strains were identified at the Mohamed V Provincial Hospital in Meknès. Furthermore, isolates from Ibn Sina Hospital’s parasitology laboratory mycology collection at CHIS Rabat were subjected to antifungal activity. Each strain was reconstituted on Mueller Hinton and Sabouraud broths using a 20% glycerol stock that was kept at −80 °C. Before usage, each strain was subcultured.

### 2.6. Determination of the Antimicrobial Activity

#### 2.6.1. Determination of the Minimum Inhibitory Concentration, Minimum Bactericidal Concentration, and Minimum Fungicidal Concentration

The minimum inhibitory concentration (MIC) was found in 96-well microplates using the reference microdilution technique [31]. Based on the amount of growth visible to the unaided eye during incubation, the minimum inhibitory concentration (MIC) of an EO is the lowest value needed to stop the tested micro-organism’s development. A stock solution of each EO produced in 10% DMSO was used for a series of dilutions to achieve concentrations of 5 to 0.93 × 10^−2^ mg/mL. These dilutions were made in Sabouraud broth for fungus and Mueller–Hinton medium for bacteria, with a final volume of 100 μL for each concentration. After this, 100 μL of microbial inoculum was added to each of the several dilution steps, with the ultimate concentrations of bacteria and fungus being 10^6^ or 10^4^ CFU/mL, respectively. Ten microliters of resazurin was applied to each well to measure the growth of bacteria after a 24 h incubation at 37 °C. The color shifted from purple to pink after a second incubation at 37 °C for two hours, indicating microbial growth. The MIC value was discovered to be the lowest concentration that stops resazurin from changing color. The growth and sterility controls were located in wells 11 and 12, respectively. This oil was used for two runs of the test. Terbinafine 250 mg, the typical antifungal employed in this study, was ground into a powder and mixed with 2 milliliters of 10% DMSO. To calculate the minimum bactericidal concentration (MBC) and minimum fungicidal concentration (MFC), 10 μL was extracted from every well that did not exhibit any growth. The samples were then plated on Mueller–Hinton (MH) agar for bacteria or in Sabouraud broth for fungi for 24 h at 37 °C. The sample concentrations that resulted in a 99.99% decrease in CFU/mL relative to the control are MBC and MFC. The MFC/MIC or MBC/MIC ratio may be used to calculate the antimicrobial efficacy of each extract. Consequently, the EO exhibits bactericidal/fungicidal activity if the ratio is less than 4 and bacteriostatic/fungistatic impact if the ratio is more than 4 [32].

#### 2.6.2. Determination of the Product Combination Behavior: Checkerboard Assays and Fractional Inhibitory Concentration Index

Using the checkerboard test, the bactericidal activities of EOs and antibiotic combinations were investigated. Using this procedure, all potential combinations between the concentration ranges of 1.2, 0.6, 0.3, 0.15, and 0.075 mg/mL were studied. The microdilution method on a microplate is another way to find the MIC of a combination (EOs/ATBs). It is associated with the lowest mixture concentrations that prevent microbial development. The FIC was computed to ascertain the impact of a combination. It serves as a gauge for a mixture’s (EOs/ATBs) effectiveness against various microbial strains [33]. For the combination of EOs (A) and ATBs (B), the calculation was carried out according to Equation (1):

(1) $$FIC_{I}=FIC_{A}+FIC_{B}=\frac{MIC_{A+B}}{MIC_{A}}+\frac{MIC_{B+A}}{MIC_{B}}$$

where *FIC_A_* is the MIC of the natural product EOs (essential oils) in the presence of commercial ATB (antibiotic) (*MIC_A+B_*) divided by the MIC of EO alone (*MIC_A_*). The *FIC_B_* is calculated by dividing the MIC of ATB B in the presence of EO A (*MIC_B+A_*) by the MIC of ATB B alone (*MIC_B_*). The recommendations set out by the European Committee on Antimicrobial Susceptibility Testing state that a FICI value of less than or equal to 0.5 signifies synergy, 0.5 to 1 additivity, >1 to 2 “no interaction” between agents, and FICI values greater than or equal to 4 indicate antagonistic effects [34].

### 2.7. Determination of the ADMET Profile and the Prediction of the Toxicity Analysis (ProTox II)

The principal EO constituents (borneol, carvacrol, (E)-caryophyllene, γ-terpinene, linalool, p-cymene, and thymol) derived from the examined oreganos were chosen for ADMET (absorption, distribution, metabolism, excretion, and toxicity) and PASS prediction tests. To choose these chemicals’ SMILES format, ChemBioDraw (PerkinElmer Informatics, Waltham, MA, USA, v13.0) [35] was utilized. Then, simulations were run utilizing the PASS-Way2Drug web prediction tool [36], in addition to the SwissADME and pkCSM web tools for ADMET prediction [37]. The term “drug-like” substances’ potential activity (Pa) and probable inactivity (Pi) are referred to as PASS [38].

A useful tool created especially for this purpose, ProTox II was utilized to examine toxicity levels and gather pertinent data on several toxicological parameters including LD50 and toxicity class [39]. With the ADMET program (SwissADME, pkCSM, and ProTox II), which was used to assess the chosen substances (ligands), the physical-chemical properties, lipophilicity, water solubility, pharmacokinetics, drug similarity, medicinal chemistry, and toxicological properties of the selected chemicals (ligands) were predicted. Credible findings on possible therapeutic uses and possible adverse effects connected to the primary chemical components found in the essential oils of the examined oreganos were achieved by employing these approaches and analytical instruments.

### 2.8. Molecular Docking

The protein targets mentioned in Table 5 have their three-dimensional structures sourced from the RCSB protein database (accessible at https://www.rcsb.org/, accessed on 13 March 2024). UCSF Chimera was utilized for the viewing of protein structure. Using Chimera software (Chimera-1.17) and Autodock Tools (version 1.5.6, The Scripps Research Institute, La Jolla, CA, USA), the protein structures were ready to be used as suitable docking targets. Protein structures were preprocessed, meaning that water molecules, heteroatoms (hetatoms), undesired protein chains, and co-crystallized ligands were removed before analysis. After adding polar hydrogen atoms and Gesteiger charges, the structures were transformed into pdbqt format so that more analyses could be performed.

After obtaining their structures from PubChem, the seven compounds’ three-dimensional structures were represented, and structural energy reduction was used. The ligands were then processed using the OpenBabel program to convert their SDF files to pdbqt formats, using the Python Prescription Virtual Screening program (AutoDock Vina).

The AutoDock Vina scoring function was used during the compound docking procedure. A grid box was placed over the protein structure once the ligand and protein molecules were chosen for docking in the Vina control. It is possible to modify the grid size under the selected active site residues before initiating the AutoDock Vina software 1.2.0 for the docking procedure. The grid box, whose dimensions and location were established using coordinates to exactly coincide with the active binding site, surrounded the search region (Table 5). PyMOL was used to analyze the properties of ligand–protein binding after the docked molecules’ resultant data, represented as free binding energy values, were collected. Removing the crystallized ligand from the protein and performing a fresh docking study in the same area where the ligand was previously present is a dependable way to confirm molecular docking. With software like PyMOL version 2.5.5, we may use this method to determine if the docked ligand overlaps with the crystalline ligand by looking at the RMSD parameter value.

### 2.9. Molecular Dynamics Simulation

GROMACS 2019.3 software was utilized to conduct molecular dynamics simulations to assess the stability of the protein–ligand combination [40]. The protein was subjected to the all-atom CHARMM36 force field, and ligand topologies were produced via the CGenFF service. The systems were neutralized by adding ions to balance the net charges after all complexes were submerged in a rectangular box filled with TIP3P water molecules. A force threshold (Fmax) of 1000 kJ/mol/nm was selected to obtain both minimal energy and maximum force using the steepest descent approach. Ensembles were kept at constant NVT and NPT (number of atoms, volume, pressure, and temperature) for molecular dynamics simulation studies. Each molecule was then subjected to 100 ns molecular dynamics simulations. In-depth knowledge of protein behavior was obtained by analyzing the files that were produced after output trajectories were formed

## 3. Results

### 3.1. Quality Control of the Investigated Oreganos’ EO

The dried plant material taken from the flowering tops of the species under study was used to compute the average essential oil output. In comparison to *O. elongatum*, *O. compactum* yielded the best EOs (Table 6). A fragrant scent and a yellowish tint define this oil.

### 3.2. GC-MS Analysis of the EOs from the Studied Oregano Species

**Figure 2:**
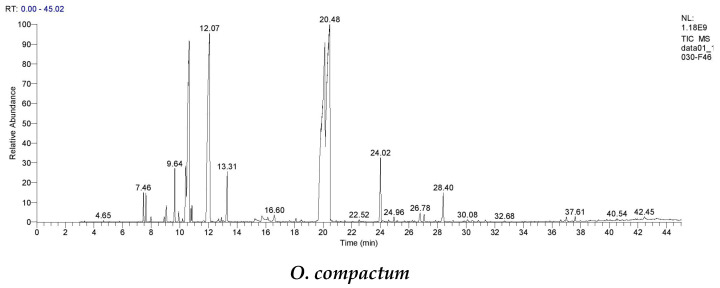

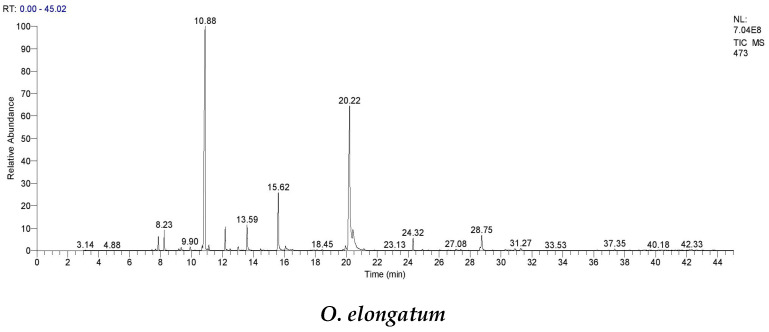
GC/MS chromatographic profiles of the EOs of the oreganos studied.

Chromatograms related to the analysis of the essential oils from the flowering tops of *Origanum compactum* and *Origanum elongatum* collected from Bouyablane and El Hamam municipalities are presented in Figure 2. These analyses allowed the deduction that both essential oils are composed of several chemical compounds in different proportions.

The chemical compositions of the EOs is shown in Table 7, from which it was determined that *O. compactum* and *O. elongatum* contain, respectively, 28 and 32 compounds, which account for an astounding 99.82% and 99.57% of their total content. The majority of the composition of the EOs in these two Origanum species is made up of oxygenated monoterpenes (46.74% and 43.71%) and monoterpene hydrocarbons (47.05% and 51.02%), respectively.

The chemical composition of *Origanum compactum* EOs is mainly composed of carvacrol (30.89%), γ-terpinene (22.89%), p-cymene (15.84%), thymol (10.21%), (E)-caryophyllene (3.63%), and linalool (2.66%), whereas *Origanum elongatum* EOs are characterized by a predominance of p-cymene (42.56%), thymol (28.43%), borneol (8.58%), linalool (3.42%), and γ-terpinene (2.98%).

**Figure 3:**
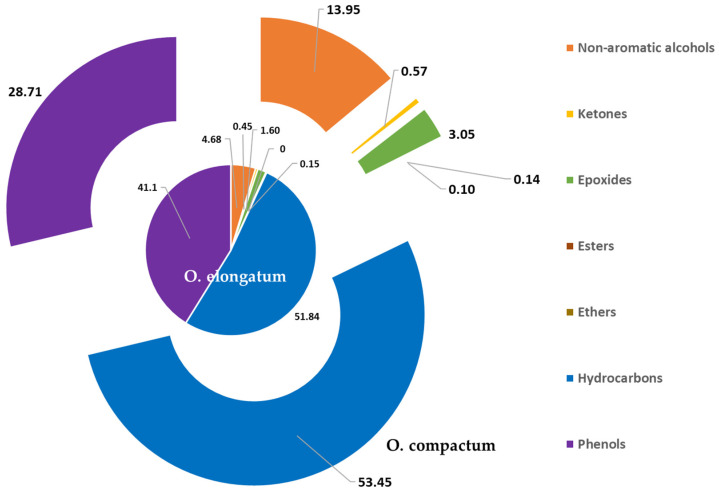
Distribution of chemical families identified in *O. elongatum* and *O. compactum* essential oils (%).

Furthermore, according to the results presented in Figure 3, we observe that the EOs of *O. compactum* and *O. elongatum* are rich in hydrocarbons (51.84% and 53.45%), phenols (41.10% and 28.71%), and non-aromatic alcohols (4.68% and 13.95%), and we also note the presence, in a low proportion, of epoxides (1.60% and 3.05%) and others (0.15% and 0.10%), respectively. In addition, low quantities of esters (0.14%) are recorded for *O. elongatum* only.

### 3.3. Antimicrobial Activity

#### 3.3.1. Study of the Sensitivity of Microbial Strains to Antibiotics and Antifungals

The antibiogram and antifungigram aim to identify and predict the sensitivity of a micro-organism to one or more antibiotics or antifungals. This sensitivity is determined with BD Phoenix and VITX 2 (Systems 9.02) for bacteria, and on the microplate for fungi. The MIC (µg/mL) measurements are presented in Table 8 and Table 9.

#### 3.3.2. Study of the Sensitivity of Microbial Strains to the Studied EO

The values of MIC, MBC, and MFC of the EOs extracted from the flowering tops of *Origanum elongatum* and *Origanum compactum* species are presented in Table 10 and Table 11.

#### 3.3.3. Antibacterial Activity of EOs from Oregano Alone

Table 10 summarizes the values of the minimum inhibitory and bactericidal concentrations of EOs from both oregano species determined against the tested bacterial strains.

Overall, both oregano EOs have shown their effectiveness against all bacteria, except for the multidrug-resistant strain of *S. aureus* STAIML/MRS/mecA/HLMUP/BLACT (*S. aureus 2220*), which showed some resistance to the evaluated EO concentrations. Gram-positive cocci showed some resistance to the evaluated EOs, except for *Streptococcus acidominimus* and *Streptococcus* group D, which are very sensitive.

Oregano essential oils exposed Gram-negative bacteria to sensitivity. All bacteria except *Enterococcus faecium* require a concentration of EOs up to 5000 µg/mL to achieve 100% inhibition, with the majority of strains being inhibited by concentrations of less than 2500 µg/mL. Additionally, it looks like the *Origanum compactum* EO is more effective than the *Origanum elongatum* EO in inhibiting the growth of Gram-positive cocci and Gram-negative bacilli.

The comparison of the MBC/MIC ratio showed that EOs exert a bactericidal action against all bacterial strains.

#### 3.3.4. Antifungal Activity of EOs from Oregano Alone

Table 11 summarizes the values of the minimum inhibitory and fungicidal concentration of EOs from the two oregano species determined against the evaluated fungal strains.

Based on the results obtained from the antifungal activity of EOs from the two oregano species, we observe that the *Origanum compactum* EO collected from Bouyablane is more potent compared to the *Origanum elongatum* EO collected from the commune of Al Hamam (Khenifra region). *Aspergillus niger* is the most sensitive strain to oregano EOs, followed by *Candida dubliniensis* and other candidoses. However, *Saccharomyces cerevisiae, Candida kyfer*, and *Candida krusei* showed some resistance to the studied EO concentrations. Despite this resistance, oregano EOs can be classified as having bactericidal and fungicidal effects.

#### 3.3.5. Antimicrobial Activity (FICI) of Oregano EOs in Combination with Antibiotics

Table 12 presents the FICI of combinations of EOs from *O. compactum* and *O. elongatum* with antibiotics (ATBs) from the checkerboard test. Among the 108 combinations of EOs studied with ATBs, 37 showed synergy (FICI < 0.5), 25 showed additivity (0.5 ≤ FICI < 1), and 44 showed no interaction (1 ≤ FICI < 2). Two combinations showed antagonistic effects (FICI > 4).

The strain that stands out the most is *Staphylococcus aureus* (*S. aureus 2220*), for which we found 14 additive and synergistic combinations. The combination of *O. elongatum* with amoxicillin (FICI = 0.375) and erythromycin (FICI = 0.281) demonstrated a synergistic impact against *A. baumannii*, which is interesting to note. A single synergistic combination of *O. compactum* and ceftriaxone (FICI = 0.375) against *E. coli* ESBL was another interesting finding. Five synergistic combinations were also observed to be effective against *E. cloacae*. Of these, *O. compactum* and tetracycline (FICI = 0.258) and *O. elongatum* and gentamicin (FICI = 0.375) were found to be particularly effective. Other combinations included imipenem (FICI = 0.281), ampicillin (FICI = 0.5), and ciprofloxacin (FICI = 0.375). These results show that these combinations may be useful in the fight against antibiotics.

Moreover, among the most significant synergistic combinations between the essential oils of the studied oregano species with antibiotics against the *K. pneumoniae* strain, four showed synergies. The findings demonstrated a strong synergy (FICI of 0.281) between *O. compactum* and amoxicillin. Further research revealed three further synergies between *O. elongatum* and the antibiotics: ciprofloxacin (FICI = 0.129), erythromycin (FICI = 0.156), and gentamicin (FICI = 0.156). The results of this study demonstrate the possibility that *O. elongatum* may increase the antibacterial activity of these drugs.

Additionally, eleven combinations showed synergistic effects against *P. aeruginosa*. Four of them were observed between *O. compactum* and imipenem (FICI = 0.250), ampicillin (FICI = 0.188), ceftriaxone (FICI = 0.188), and erythromycin (FICI = 0.141). The other seven combinations were recorded for *O. elongatum* with imipenem (FICI = 0.375), ampicillin (FICI = 0.066), ciprofloxacin (FICI = 0.250), ceftriaxone (FICI = 0.125), erythromycin (FICI = 0.078), tetracycline (FICI = 0.094), and vancomycin (FICI = 0.313).

### 3.4. PASS, ADME-Tox, and the Estimation of the Effectiveness of Possibly Active Components Extracted from the Examined Oregano EO

The websites pkCSM and SwissADME aid in understanding the pharmacokinetic properties and drug-likeness of certain compounds. All of the chosen compounds’ lipophilicity ratings indicated that they were quite soluble in water (Table 13). The chosen substances have good skin permeability values (log Kp) and strong Caco-2 permeability values. Hence, the majority of drugs have excellent intestine absorption (HIA > 30%). P-glycoprotein, or P-gp, is necessary for the distribution and absorption of drugs. None of the investigated chemicals operate as P-gp I and P-gp II inhibitors, nor are the main constituents of the two essential oils under investigation P-gp substrates. All of the isolated compounds from the essential oils under study had a CNS score > −3.0 and a log BB > 0.3, suggesting their ease of passage over the blood–brain barrier (BBB) and weak penetration of the central nervous system (CNS). Within tissues, their distribution volumes (logVDss) vary from 0.152 L/kg to 0.697 L/kg. The primary constituents of the examined essential oils are unlikely to have negative effects when taken orally due to drug interactions; nonetheless, cytochrome P450 (CYP) enzymes and molecular interactions are crucial for medication clearance. Retinal organic cation transporters 2 (OCT2) and hepatic and renal substrates’ total clearance (CLTOT) were represented as log mL/min/kg anticipated to forecast the excretion pathway. All of the phytochemical elements under study had a good overall clearance value and were excretable, according to the data.

To evaluate the possible toxicity of *O. compactum* and *O. elongatum* essential oils in terms of AMES, hepatotoxicity, hERG potassium channel inhibition, skin sensitization, and cytotoxicity, the phytochemical components of these oils were investigated (Table 14). With the possible exception of carvacrol and thymol compounds, which may have very little hepatic effects, the data demonstrate the lack of cytotoxicity and liver toxicity effects.

### 3.5. Molecular Docking

For molecular docking investigations, chemicals discovered via CG/MS were used to identify the essential oils of *O. compactum* and *O. elongatum*, which have demonstrated antibacterial activity and strong synergy with antibiotics in vitro. Using molecular docking studies, the antibacterial properties of the compounds were inferred together with their probable mechanism of action on the target proteins 1JZQ, 2VEG, 2ZDQ, 3RAE, 3SRW, 3UDI, 1KZN, 5J8G, 5OE3, and 4URN. To assess the impacts of different compounds isolated from *O. compactum* and *O. elongatum* on their particular activities, molecular docking simulations were run on a variety of systems. The docking results for the top-ranked compounds were prioritized based on their binding score, revealing remarkable interaction patterns with the selected protein targets (Table 15).

**Figure 4:**
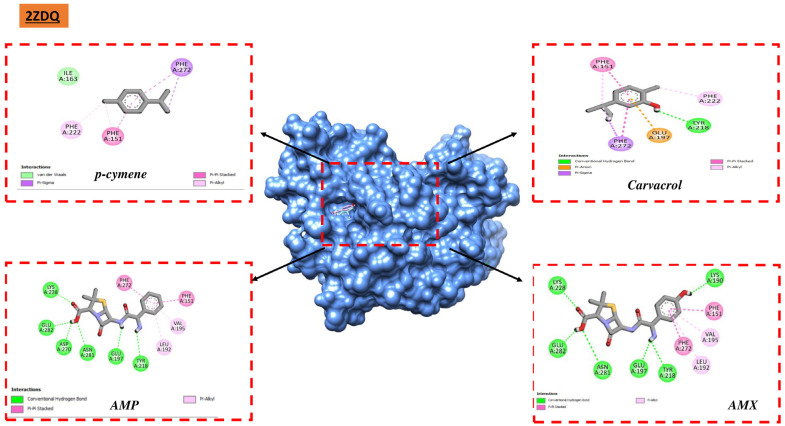
Interaction of 2ZDQ with ligands.

Certain chemicals including carvacrol, thymol, p-cymene, γ-terpinene, and (E)-caryophyllene form substantial interactions with different protein targets, according to the examination of the docking data. This study specifically emphasized the exceptional affinity of p-cymene and carvacrol with important proteins like D-alanine ligase. The compounds exhibited the capacity to establish stable and resilient complexes with D-alanine ligase, indicating favorable possibilities for their possible engagement in modifying these particular systems. These substances may be good candidates for the creation of antibacterial medications that target D-alanine ligase, as this enzyme is essential to the growth and maintenance of bacterial cell walls. Moreover, it is significant that D-alanine ligase has a strong binding affinity for all investigated antibiotics, particularly ampicillin and amoxicillin (Figure 4).

**Figure 5:**
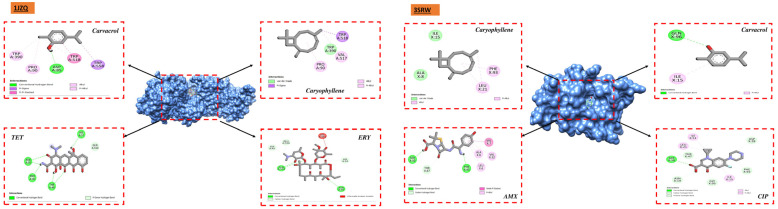

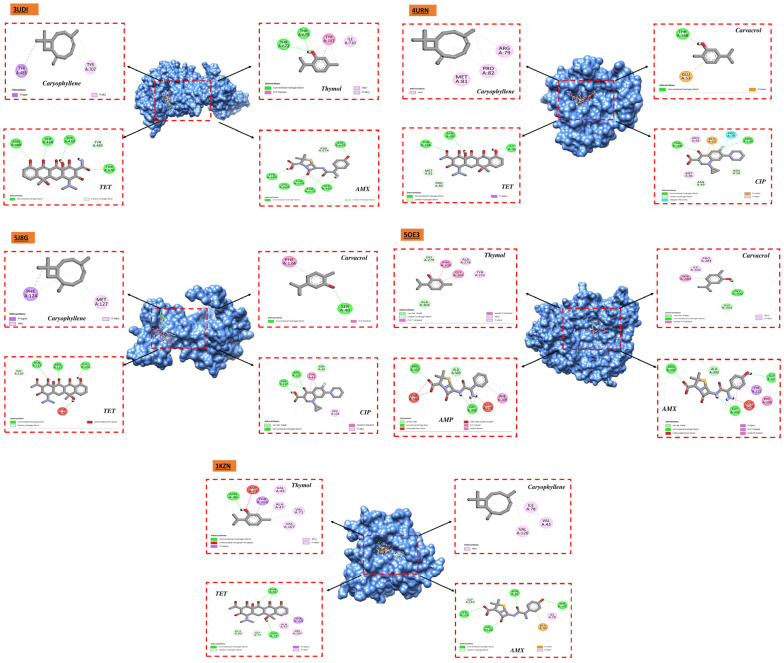
Interaction of target proteins with ligands having the highest binding affinities.

Thymol also showed a strong affinity for the N-terminal domain structures of PqsA in complexes with anthraniloyl-AMP and 6-fluoroanthraniloyl-AMP and the Penicillin-binding protein 1a (PBP1a) protein. These findings emphasize thymol’s capacity to form significant interactions with these molecular targets and show its potential for inhibition within the framework of these particular systems. These findings raise additional questions about thymol’s possible application in the development of tailored treatments by indicating that it may have a significant role as an inhibitory agent in the context of processes connected to these particular molecular targets. The protein targets isoleucyl-tRNA synthetase, dihydropteroate synthase, D-alanine ligase, dihydrofolate reductase, *E. coli* crystalline structure, Staph crystalline structure, *E. cloacae* nitroreductase structure, and Penicillin-binding protein 1a (PBP1a) protein have all been found to have a strong binding affinity with (E)-caryophyllene. These findings demonstrate caryophyllene’s capacity to assemble strong, stable complexes with a variety of proteins, highlighting its potential for modifying these particular systems. Furthermore, the residue interactions between the target proteins and the ligands (antibiotics and chemicals from essential oils) under study are shown in Figure 5 below.

The main substances analyzed form connections that support the stabilization of the ligand–protein complex, improving binding affinity. Stronger and more specific binding between the ligand and the protein is promoted by specific interactions like well-positioned hydrogen bonds, suitable electrostatic interactions, and compatible hydrophobic regions. This could have a positive effect on the compound’s efficacy in modulating the biological systems under study.

### 3.6. Molecular Dynamics

For MD simulation, the optimal protein–ligand complexes were chosen based on the outcomes of molecular docking. The molecular docking study and the PASS biological activity prediction accord well. We checked the simulation trajectory data for hydrogen bonding, radius of gyration (Rg), root mean square fluctuation (RMSF), and root mean square deviation (RMSD). In the examined essential oils, thymol, and (E)-caryophyllene were shown to have the best binding energies with the target proteins. Thus, during 100 ns MD simulations, the complexes 5J8G + caryophyllene, 3UDI + thymol, 1JZQ + caryophyllene, 1KZN + caryophyllene, and 1KZN + thymol were chosen.

#### 3.6.1. Structural Dynamics of 1KZN

**Figure 6:**
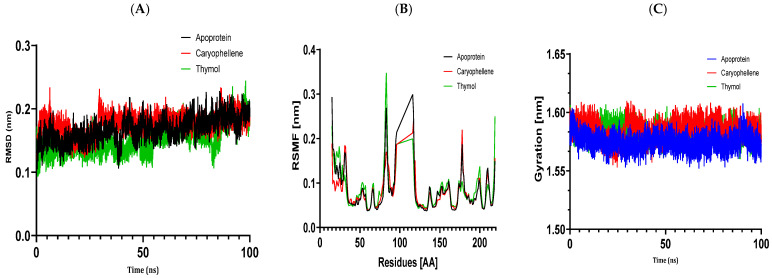
Structural dynamics of the 1KZN protein. (**A**) RMSD. (**B**) RMSF. (**C**) Radius of gyration.

The complexes of the DNA gyrase protein (1KZN) with thymol and caryophyllene compounds were investigated using molecular dynamics simulations (Figure 6). We computed the root mean square deviation (RMSD) to evaluate these complexes’ stability. The RMSD of the protein fluctuated within an average range of 0.17 nm. More stable protein structures are correlated with lower RMSD values. We looked at RMSF diagrams to find ligand-bound states. The average RMSF of protein 1KZN was 0.08 nm. Nonetheless, thymol’s and caryophyllene’s average RMSD values decreased, suggesting that the complexes remained stable throughout the experiment. An indication of the protein’s structural compactness can be found in the radius of gyration (Rg). In the case of the complexes, the average Rg values remained practically unchanged or slightly decreased, indicating that the ligands did not significantly alter the structural compactness of protein 1KZN (Figure 6).

#### 3.6.2. Structural Dynamics of 5J8G

**Figure 7:**
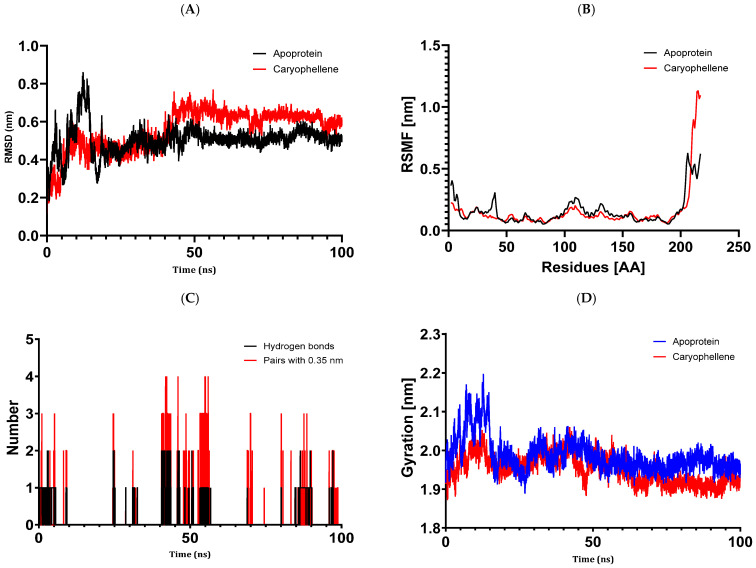
Structural dynamics of the 5J8G protein: (**A**) RMSD. (**B**) RMSF. (**C**) Total number of intramolecular hydrogen bonds. (**D**) Radius of gyration.

We studied protein 5J8G in detail, which is a representation of the structure of *E. cloacae* nitroreductase attached to para-nitrobenzoic acid. A balanced simulation was demonstrated by the dynamic variations in the structure of the 5J8G + caryophyllene complex that were observed (Figure 7). Throughout the 100 ns simulation, protein 5J8G’s RMSD fluctuations stayed quite near to the thermal average. Furthermore, caryophyllene’s heavy atoms’ RMSD values showed how stable it was in comparison to the protein; they were significantly lower than the protein’s, indicating that caryophyllene stayed close to its original binding location. Less than 3 Å was found to be the average RMSF fluctuation during a 100 ns period, suggesting a robust interaction between caryophyllene and protein 5J8G without a discernible change in the protein structure caused by caryophyllene. Moreover, caryophyllene attached to the 5J8G active site with an average of 0.14 hydrogen bonds and 0.13 pairs across a 0.35 nm radius, according to hydrogen bond analysis.

#### 3.6.3. Structural Dynamics of 3UDI

**Figure 8:**
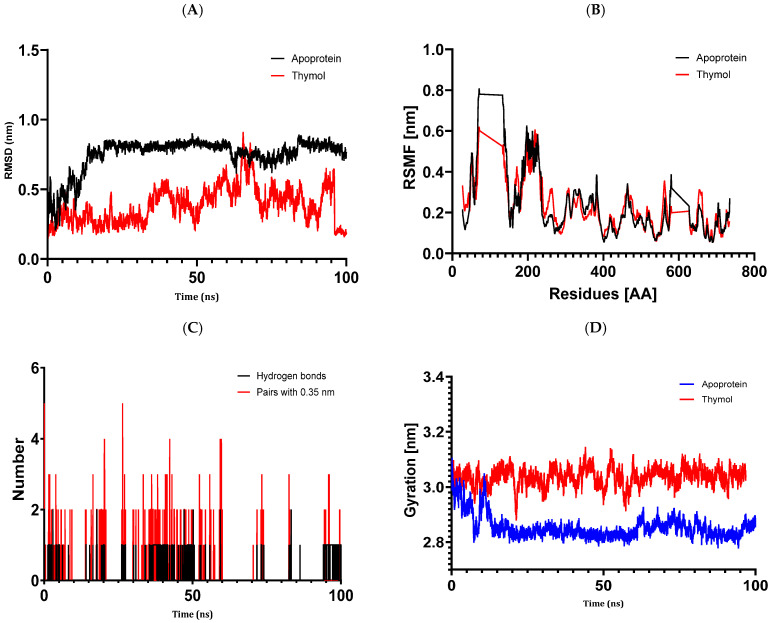
Structural dynamics of the 3UDI protein: (**A**) RMSD. (**B**) RMSF. (**C**) Total number of intramolecular hydrogen bonds. (**D**) Radius of gyration.

Our study’s findings demonstrate a balanced simulation of the 3UDI + thymol complex, with variations in the 3UDI protein’s root mean square deviation staying quite near to the thermal average throughout the simulation’s 100 ns (Figure 8). Additionally, the RMSD values of thymol’s heavy atoms show how stable it is in comparison to the protein; they are noticeably lower than the protein’s, suggesting that thymol stayed securely fixed to its original binding site. Without appreciably changing the structure of the protein, the root mean square fluctuations throughout a 100 ns time stay below 3 Å, demonstrating a tight connection between thymol and the 3UDI protein. Thymol binds to the active pocket of 3UDI firmly, creating an average of 0.11 hydrogen bonds and 0.10 pairs within a radius of 0.35 nm, according to hydrogen bond studies. Following the interaction of the 3UDI protein with thymol, the radius of gyration values varied within a tight range of 2.75 to 3.2 Å until the simulation’s conclusion, indicating the protein’s high flexibility. These results demonstrate a persistent contact that thymol maintains with the binding pockets of 3UDI, while also highlighting a strong relationship between thymol and the crystalline structure of Acinetobacter baumannii PBP1a in association with penicillin G, without appreciably altering the protein’s shape.

#### 3.6.4. Structural Dynamics of 1JZQ

**Figure 9:**
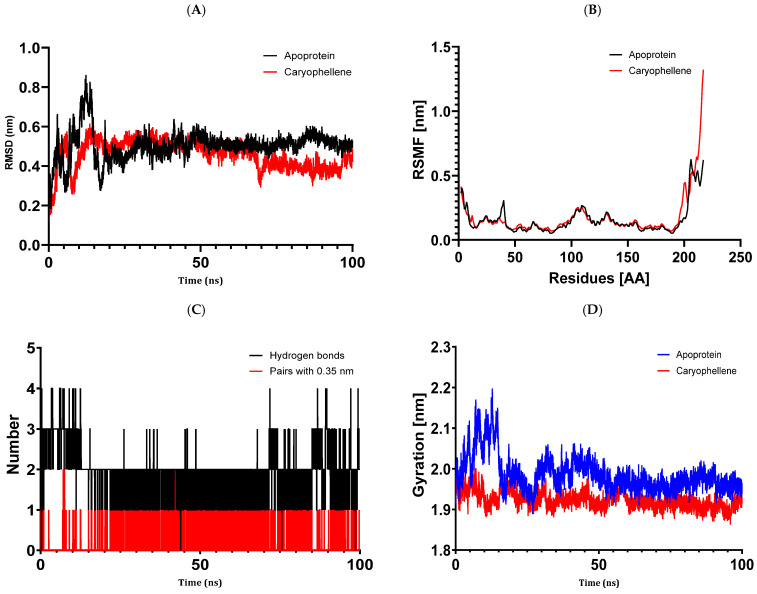
Structural dynamics of the 1JZQ protein: (**A**) RMSD. (**B**) RMSF. (**C**) The total number of intramolecular hydrogen bonds. (**D**) Radius of gyration.

The study we conducted on the protein 1JZQ complexed with caryophyllene demonstrates that the simulation was balanced and that, across the 100 ns of simulation, fluctuations in the root mean square deviation were pretty near to the thermal average (Figure 9). This is acceptable for small globular proteins. Caryophyllene’s stability in the protein is further demonstrated by the RMSD values of its heavy atoms, which suggest that the compound stayed near its original binding site. Protein 1JZQ and caryophyllene exhibit a strong binding without creating major changes in the protein’s structure, as indicated by the RMSF values, which show that all fluctuations were below 3 Å. Caryophyllene attaches to the active site of 1JZQ with an average of 1.98 hydrogen bonds and 0.14 pairs across a radius of 0.35 nm, according to the hydrogen bonding study as well. After interacting with caryophyllene, protein 1JZQ exhibited good flexibility, as seen by the radius of gyration values fluctuating within a narrow range of 1.85 to 2.2 Å until the simulation’s conclusion. These findings imply that protein 1JZQ and caryophyllene have a strong interaction that does not significantly alter the protein’s structure.

The molecular dynamics simulation results corroborate findings from molecular docking and earlier in vitro studies. The ligands under examination exhibit the highest level of inhibitory activity and form stable connections with certain amino acids present in the target proteins. Remarkably, these interactions exhibit little variations in their parameters throughout the 100 ns simulation time.

## 4. Discussion

As one of the top three health issues identified by the World Health Organization, antibiotic resistance has become a major factor in worldwide mortality in the twenty-first century [41]. It happens when bacteria find methods to resist the effects of antibiotics; this is a problem that is made worse by the overuse and poor application of antibiotics in agricultural and medical settings [42,43]. Since the 1940s, when modern antibiotics became widely available, the problem has become worse, and almost all bacterial types now exhibit some degree of resistance. Therefore, it is imperative to quickly ascertain alternative materials capable of efficiently suppressing bacteria that have developed resistance to carbapenems. These include methicillin-resistant *Staphylococcus aureus* (MRSA), *Acinetobacter baumannii, Enterobacteriaceae* (especially *Klebsiella pneumoniae*, *Escherichia coli*, and *Enterobacter* spp.), and *Pseudomonas aeruginosa*.

The quest for efficacious antimicrobial therapies has conventionally centered on using the therapeutic characteristics of naturally occurring compounds derived from a wide array of organisms, including bacteria, fungi, plants, algae, and mammals. Exploring plant-based bioactive molecules as possible alternatives to conventional antibiotics has gained traction in recent times. The antibacterial qualities of essential oils, for example, have been the focus of much research due to their ability to combat common human diseases [44,45]. Their antibacterial capabilities can be strengthened by mixing them with other substances, such as essential oils and medicines [46]. To determine the effectiveness of *O. compactum* and *O. elongatum* essential oils in preventing infection, we developed cultures of bacteria that were resistant to several drugs. We then exposed these strains to the essential oils alone and in combination with antibiotics.

The essential oils under examination underwent a thorough investigation using GC-MS, which revealed their unique chemical signatures. Notably, *O. compactum* essential oil showed trace components, including €-caryophyllene and linalool together, with a substantial presence of beneficial compounds like carvacrol, γ-terpinene, p-cymene, and thymol. On the other hand, the main constituents of *O. elongatum* essential oil were p-cymene, thymol, and borneol. These results are consistent with earlier studies on the topic.

Numerous research studies examining the characteristics of *O. compactum*’s flowering tops have focused on Morocco. Two studies, in particular, have drawn notice due to their findings. In 2018, Laghmouchi and colleagues conducted an analysis of fourteen essential oils collected from six distinct zones in northern Morocco, spanning diverse geographic areas [26]. The findings showed considerable quantities of p-cymene (6.69 to 42.64%), thymol (0.16 to 34.29%), and γ-terpinene (2.95 to 22.97%), followed by a high concentration of carvacrol (range from 2.18 to 63.65%). Building on this understanding, a follow-up research study was carried out in 2021 by Ez-zriouli and associates [47]. Their findings demonstrated the prevalence of carvacrol (72.97%) in *O. compactum* essential oil together with significant concentrations of p-cymene (14.5%) and γ-terpinene (6.01%). However, in other countries, several studies have shown that *O. compactum* essential oil is characterized by a predominance of carvacrol [48,49,50,51,52].

A recent Moroccan research study discovered that *O. elongatum* essential oil exists in four chemical forms: carvacrol, carvacrol/thymol, carvacrol/p-cymene, and thymol [49,53,54]. We detected the p-cymene/thymol/borneol form in our investigation. Given that p-cymene is a precursor in the biosynthesis pathways of both carvacrol and thymol, it is plausible that this variance results from harvesting at a different time.

The emergence of antibiotic-resistant bacterial pathogens has rendered most available antibiotics ineffective [55]. Alternative strategies are therefore necessary to combat drug-resistant bacterial infections. Combined therapies between EOs and conventional antibiotics to enhance their effectiveness appear to be the most effective solution. Indeed, oregano species’ essential oils have shown significant antibacterial activity against all tested bacteria. It is noteworthy that *O. compactum* essential oil exhibited the highest antibacterial efficacy, primarily attributable to the high levels of its bioactive constituents, including Carvacrol, γ-Terpinene, p-Cymene, Thymol, and (E)-Caryophyllene. These substances have been praised for their antibacterial properties because of their distinct chemical composition and strong synergy when combined [56,57,58].

Rosato, Scandorieiro, Oumam, and colleagues concluded that oregano essential oils act against bacteria (Gram-positive and Gram-negative) and fungi (yeasts and molds), including multidrug-resistant strains [59,60,61]. In our study, Gram-negative bacteria showed greater sensitivity to both evaluated oregano species than Gram-positive bacteria and fungi, corroborating the results of Lambert’s and Amakran’s studies [62,63]. The different sensitivity profiles of the tested strains could be justified by the structure of the cell wall.

Essential oils and antibiotics work synergistically, which is one of the newest tactics to tackle bacterial resistance [10]. When two substances work better together than they do apart, this is known as a synergistic effect. It also occurs when the observed inhibition of a combination is greater than the expected inhibitions of the individual compounds. The presence of multiple active antibacterial constituents and interactions between different components of both oils are responsible for this synergy phenomenon. These interactions can disrupt the internal membranes of bacteria, increasing the permeability of structures, inhibiting microbial motility, inhibiting microbial ATPase, and/or inhibiting efflux pumps. They can also increase the solubility and/or availability of one or more oil constituents or act on different targets, resulting in enhanced antibacterial effects [64].

This study has revealed synergistic interactions between oregano essential oils and selected antibiotics. The works of Wendy et al., 2012 [14,65,66,67], on the use of Origanum essential oils in combination with antibiotics have shown significant synergistic interactions. Our results support these studies by showing that Origanum essential oils have a synergistic effect on antibiotic-resistant bacteria, such as *Klebsiella pneumoniae, Escherichia coli, Pseudomonas aeruginosa, Staphylococcus aureus, and Acinetobacter baumannii*, which are frequently responsible for infections linked to healthcare settings. Moreover, the correlation between the composition profile and antibacterial activity can elucidate the synergistic effect. This was especially noticeable when *O. compactum* and *O. elongatum* essential oils were combined with antibiotics, suggesting that the concentration of monoterpenes in the studied essential oils may be related to their potency.

The FICI values obtained in our study on the 108 interactions examined in vitro, as well as the strong binding affinities to the protein targets studied in silico, reveal promising synergies in the use of oregano essential oils in combination with different antibiotics. When combined with ampicillin against *P. aeruginosa*, amoxicillin against strains of *S. aureus, A. baumannii, and K. pneumoniae*, and ciprofloxacin against *P. aeruginosa, E. cloacae*, and *K. pneumoniae*, it has been observed that the essential oils of *O. elongatum* and *O. compactum* are highly effective. Furthermore, when coupled with the essential oils, ceftriaxone has demonstrated encouraging synergistic benefits against *P. aeruginosa* and *E. coli* ESBL. Additionally, when combined with tetracycline, the essential oils elicit synergistic activity against strains of *E. cloacae, P. aeruginosa*, and *S. aureus*. Furthermore, the results of molecular docking demonstrate a noteworthy affinity of these optimal combinations, particularly with EOs high in (E)-caryophyllene and thymol. Our investigation demonstrated a considerable increase in antibacterial activity when ampicillin was combined with the essential oils of *O. compactum* and *O. elongatum*, with FICI values of 0.188 and 0.066, respectively. Recent research has demonstrated that a potent combination of ampicillin and oregano essential oil is very successful in treating lung infections brought on by bacteria that are resistant to drugs [68,69]. The mechanism of action of this potent medication is to directly target and inhibit the transpeptidase enzyme, which is in charge of binding the essential peptidoglycan molecules that makeup bacteria’s cell walls. Ampicillin interferes with this process, making the cell wall brittle and weak, which eventually leads to its rupture. Studies have shown that some essential oils work well when paired with amoxicillin, in addition to displaying encouraging fractional inhibitory concentration index values. *O. compactum* and *O. elongatum* had FICI values of 0.125 and 0.123, respectively, against *S. aureus*. On the other hand, *O. elongatum* showed a FICI of 0.375 against *A. baumannii*, and *O. compactum* demonstrated a FICI of 0.281 against *K. pneumoniae*. Amoxicillin works by binding to the transpeptidase enzyme, which breaks the bonds between peptidoglycan molecule-essential components of the bacterial cell wall. Its stability in the acidic environment of the stomach further increases its effectiveness when taken orally. Because of its decreased vulnerability to degradation by beta-lactamase enzymes, it is a more effective treatment for bacteria resistant to antibiotics [70]. These findings demonstrate how effective essential oils may be used in tandem with medicines to treat bacterial illnesses. Regarding the FICI against *P. aeruginosa* (FICI = 0.250), *E. cloacae* (FICI = 0.375), and *K. pneumoniae* (FICI = 0.129), the combination of *O. elongatum* essential oil and ciprofloxacin has shown promising results. The drug ciprofloxacin works by blocking DNA gyrase and topoisomerase IV. It is used for some bacterial infections, including respiratory, urinary, gastrointestinal, and skin infections. Ciprofloxacin inhibits these enzymes’ ability to operate through their interactions, which causes DNA supercoils to accumulate. Compounds present in *O. elongatum* essential oils and the antibiotic ciprofloxacin work together to impair DNA repair, transcription, and replication forks, making it impossible for the bacterium to proliferate and survive. While the specific reaction of bacterial cells to fluoroquinolone exposure is still not fully understood, a DNA-damage-induced SOS response is thought to be important [71,72]. When combined with ceftriaxone, oregano EO has demonstrated encouraging FICI values against *P. aeruginosa* (FICI = 0.188 and 0.125 for *O. elongatum* and *O. compactum*, respectively) and *E. coli* ESBL (FICI = 0.375 for *O. compactum*). This partnership suggests that treating bacterial illnesses may be more successful. Ceftriaxone is a more potent alternative against bacterial strains resistant to antibiotics due to its higher stability when compared to first- and second-generation cephalosporins [73]. It can also be used to treat infections of the central nervous system due to its capacity to cross the blood–brain barrier. Furthermore, it differs from other cephalosporins that are usually only administered intravenously in that they can be administered intramuscularly or intravenously [74]. The effectiveness of the combination of oregano essential oils (EOs) with tetracycline was studied against different multidrug-resistant bacterial strains. The results obtained revealed promising values of the FICI, highlighting significant synergies between oregano EOs and tetracycline. The found FICI value for *P. aeruginosa* (*O. elongatum*) is 0.094, 0.258 for *E. cloacae* (*O. compactum*), and 0.313 and 0.490 for *S. aureus* (*O. compactum* and *O. elongatum*), respectively. To stop messenger RNA from being converted into essential proteins, tetracycline binds to the ribosomal RNA of the bacterium. This inhibition gives tetracycline a bacteriostatic property, preventing bacterial growth without directly killing them, due to its ability to penetrate cell membranes because of its lipophilicity. Furthermore, tetracycline demonstrates particular efficacy against bacteria-producing beta-lactamase enzymes capable of breaking down penicillin antibiotics [75].

Studies utilizing molecular docking are widely utilized to ascertain the possible interaction between a protein and a ligand. These investigations seem to be beneficial in revealing significant information on the antibacterial qualities of natural sources. They also provide important insights into the complex interactions and possible mechanisms of action at the binding sites of various bacterial proteins [76]. We carried out docking analyses on the main constituents of the essential oils of the two oregano species under investigation to better comprehend these mechanisms. The receptors that we selected for our study were 1JZQ, 2VEG, 2ZDQ, 3RAE, 3SRW, 3UDI, 1KZN, 5J8G, 5OE3, and 4URN. These essential chemicals are vital in stabilizing the ligand–protein complex and eventually raising binding affinity through the establishment of connections. The compound’s ability to regulate biological systems is ultimately improved by the specific arrangement of hydrogen bonds and corresponding hydrophobic areas, which promotes a deeper and more focused link between the ligand and protein. Furthermore, there may be increased potential for permeability [77], absorption, and bioavailability in substances with smaller molecular weights, less lipophilicity, and decreased hydrogen bonding capacity [78,79].

The results of molecular docking investigations and in vitro trials are further validated by the results of molecular dynamics simulations conducted on complex models, such as 5J8G + caryophyllene, 3UDI + thymol, 1JZQ + caryophyllene, 1KZN + caryophyllene, and 1KZN + thymol. This demonstrates the significance of these investigations. They validate that caryophyllene and thymol interact steadily with target proteins, bolstering the theory that these substances are the most potent inhibitors. In light of the possible creation of novel therapeutic antimicrobial medicines, our data provide credence to the theory that these chemicals form dynamically stable complexes throughout 100 ns simulations.

The main constituents of an essential oil as well as the mixture of all of its constituents determine how resistant bacteria are to antimicrobial agents. An essential oil’s ability to combat germs is directly related to its chemical makeup, the main compounds’ functional groups, and the way these components work together. This synergy can arise from several processes, including the inhibition of metabolic pathways or the agents involved, as well as the disruption of the bacterial cell membrane, which makes it easier for additional antibacterial agents to work against the bacterium [80,81].

Based on the findings of molecular docking studies and molecular dynamics (MD) simulations, it is possible to explain the potential mechanism of action of compounds from *Origanum compactum* and *Origanum elongatum* essential oils (EOs), such as thymol, (E)-caryophyllene, carvacrol, p-cymene, borneol, and linalool, with antibiotics against the multidrug-resistant bacterial strains (*K. pneumoniae, E. coli, E. cloacae, P. aeruginosa, S. aureus,* and *A. baumannii*) that are studied.

According to molecular docking studies, thymol, carvacrol, (E)-caryophyllene, and other EO components have substantial interaction with a variety of bacterial protein targets. For instance, D-alanine ligase, an enzyme required for the construction of bacterial cell walls, has demonstrated a significant affinity for carvacrol and p-cymene. The (E)-caryophyllene has demonstrated great affinity with many protein targets, including isoleucyl-tRNA synthetase and dihydropteroate synthase, whereas thymol has demonstrated considerable affinity with protein PBP1a. These interactions imply that these substances may block these proteins, interfering with processes that are necessary for the survival of bacteria.

Molecular dynamics simulations have confirmed the stability of the complexes formed between essential oil compounds and target proteins. For example, in the case of the 1KZN + caryophyllene complex involving the DNA gyrase protein, the root means square deviation (RMSD) fluctuations of the protein remained stable throughout the simulation, indicating a robust interaction between caryophyllene and the protein. The root means square fluctuations and radius of gyration values also remained within acceptable ranges, confirming the stability of the protein–ligand complex. This work demonstrates the important impact that essential oils have on bacterial cells because of their hydrophobic characteristics, which increase the permeability of the cells and cause cytoplasmic components to seep out. Remarkably, some other pathways have also been discovered, such as cell wall breakdown, ATP generation interference, pH level disruption, suppression of protein synthesis, and even DNA damage [82,83]. These results point to a potential relationship between the antibiotics under investigation and oregano essential oils, either by focusing on a common mechanism or by making the target location easier to reach.

## 5. Conclusions

This study investigated the potential use of essential oils from the flowering tops of *O. compactum* and *O. elongatum* in treating diseases brought on by drug-resistant bacteria. There were significant variances in the wide variety of chemical structures exhibited by both essential oils. *O. elongatum* essential oil stood out for having a lot of p-cymene and thymol, whereas *O. compactum* essential oil had large quantities of carvacrol. The results demonstrated the strong antibacterial qualities of these essential oils against resistant bacteria, such as *Acinetobacter baumannii, Pseudomonas aeruginosa, Escherichia coli, Klebsiella pneumoniae*, and *Staphylococcus aureus*. Their high content of potent substances, including carvacrol, thymol, and (E)-caryophyllene, is mostly to blame for this. These essential oils show remarkable synergistic benefits when used with conventional antibiotics, especially against Gram-negative bacteria. There was significant synergy between the antibiotics ampicillin, amoxicillin, ciprofloxacin, ceftriaxone, and tetracycline. The isolated molecules from essential oils also showed encouraging pharmacokinetic qualities, indicating that they may be used in medicine. These substances astonishingly demonstrated the capacity to cross the blood–brain barrier, in addition to their low toxicity. Intriguingly, studies conducted in silico have shown that the substances included in essential oils establish robust connections with bacterial proteins that are important for vital functions, including DNA replication, cell wall building, and antibiotic resistance. These interactions could explain the synergistic improvements in antibiotic efficacy that were seen. An approach that is very encouraging in the fight against drug-resistant bacteria is the use of essential oils of *Origanum compactum* and *Origanum elongatum* rather than conventional antibiotics. These oils’ cooperative activity suggests great potential for novel therapeutic opportunities. This is explained by their distinct chemical makeup and capacity to interact with certain bacterial proteins. To confirm the safety and therapeutic efficacy of these synergistic effects, more research is required. Furthermore, maintaining essential oils’ ability to effectively treat antibiotic resistance over time depends on their careful and controlled use.

## Figures and Tables

**Table 1:** Distribution of individuals in the populations of the oregano species studied and their harvest sites by region.

Latin Name	Abbreviation	Harvest Site		Parts Used	Latitude (x)	Longitude (y)	Altitude (m)	Harvest Year
		Region	Locality					
*Origanum compactum*	*O. compactum*	Taza	Bouyablane	Flowering tops	33°41′31″ N	4°03′43″ W	3192	July 2022
*Origanum elongatum*	*O. elongatum*	Khenifra	El hammam		33°10′28″ N	5°28′09″ W	1125	

**Table 2:** The taxonomic classification of the genus *Origanum*.

Reign	Plants
Kingdom	Plantae
Division	Magnoliophyta
Class	Magnoliopsida
Order	Lamiales
Family	*Lamiaceae*
Genus	*Origanum*

**Table 3:** List of antibiotics studied.

Antibiotics (ATBs)	Abbreviation	Chemical Family
Gentamycin	GEN	Aminoglycosides
Vancomycin	VAN	Glycopeptides
Amoxicillin	AMX	Beta-lactams
Ampicillin	AMP	
Ceftriaxone	CRO	
Ciprofloxacin	CIP	
Imipenem	IMP	
Erythromycin	ERY	Macrolides
Tetracycline chlorhydrate	TET	Tetracyclines

**Table 4:** List of tested bacterial and fungal strains, together with references.

Strains		Abbreviations	References
Gram-positive cocci	*Staphyloccocus epidermidis*	*S. epidermidis*	5994
	*Staphyloccocus aureus BLACT*	*S. aureus BLACT*	4IH2510
	*Staphyloccocus aureus STAIML/MRS/mecA/HLMUP/BLACT*	*S. aureus 2220*	2DT2220
	*Streptococcus acidominimus*	*S. acidominimus*	7DT2108
	*Streptococcus group D*	*S. group D*	3EU9286
	*Streptococcus agalactiae*	*S. agalactiae (B)*	7DT1887
	*Streptococcus porcinus*	*S. porcinus*	2EU9285
	*Enterococcus faecalis*	*E. faecalis*	2CQ9355
	*Enterococcuss faecium*	*E. faecium*	13EU7181
Gram-negative bacilli	*Acinetobacter baumannii*	*A. baumannii*	7DT2404
	*Acinetobacter baumannii*	*A. baumannii 2410*	7DT2410
	*Escherichia coli*	*E. coli*	3DT1938
	*Escherichia coli ESBL*	*E. coli ESBL*	2DT2057
	*Escherichia coli ESBL*	*E. coli ESBL 5765*	2DT5765
	*Enterobacter aerogenes*	*E. aerogenes*	07CQ164
	*Enterobacter cloacae*	*E. cloacae*	02EV317
	*Enterobacter cloacae*	*E. cloacae 2280*	2DT2280
	*Citrobacter koseri*	*C. koseri*	3DT2151
	*Klebsiella pneumoniae* ssp. *pneumoniae*	*K. pneumoniae*	3DT1823
	*Klebsiella pneumoniae* ssp. *pneumoniae*	*K. pneumoniae 1015*	3DT1015
	*Proteus mirabilis*	*P. mirabilis*	2DS5461
	*Pseudomonas aerogenosa*	*P. aerogenosa*	2DT2138
	*Pseudomonas aerogenosa*	*P. aerogenosa 1124*	2DT1124
	*Pseudomonas fluorescence*	*P. fluorescence*	5442
	*Pseudomonas putida*	*P. putida*	2DT2140
	*Serratia marcescens*	*S. marcescens*	375BR6
	*Salmonella* sp.	*Salmonella* sp.	2CG5132
	*Shigella* sp.	*Shigella* sp.	7DS1513
	*Yersinia enterocolitica*	*Y. enterocolitica*	ATCC27729
Yeasts	*Candida albicans*	*C. albicans*	Ca
	*Candida kefyr*	*C. kefyr*	Cky
	*Candida krusei*	*C. krusei*	Ckr
	*Candida parapsilosis*	*C. parapsilosis*	Cpa
	*Candida tropicalis*	*C. tropicalis*	Ct
	*Candida dubliniensis*	*C. dubliniensis*	Cd
	*Saccharomyces cerevisiae*	*S. cerevisiae*	Sacc
Fungi	*Aspergillus niger*	*A. niger*	AspN

**Table 5:** Protein targets and molecular docking parameters.

Protein	PDB ID	Grid Box Center Coordinates	Grid Box Size
Isoleucyl-tRNA synthetase	1JZQ	center_x = −27.803	size_x = 34
		center_y = 6.619	size_y = 21
		center_z = −28.722	size_z = 23
DNA gyrase	1KZN	center_x = 18.325	size_x = 20
		center_y = 30.783	size_y = 38
		center_z = 36.762	size_z = 38
Dihydropteroate synthase	2VEG	center_x = 31.404	size_x = 24
		center_y = 48.530	size_y = 24
		center_z = 0.204	size_z = 18
D-Alanine ligase	2ZDQ	center_x = 47.378	size_x = 23
		center_y = 12.782	size_y = 26
		center_z = 5.730	size_z = 32
Type IV topoisomerase	3RAE	center_x = −33.300	size_x = 32
		center_y = 67.893	size_y = 25
		center_z = −23.840	size_z = 24
Dihydrofolate reductase	3SRW	center_x = −4.701	size_x = 26
		center_y = −31.536	size_y = 28
		center_z = 6.341	size_z = 23
Penicillin-binding protein 1a PBP1a	3UDI	center_x = 34.198	size_x = 24
		center_y = −1.249	size_y = 24
		center_z = 12.715	size_z = 28
Crystal structure of Staph ParE 24 kDa	4URN	center_x = −31.684	size_x = 28
		center_y = 8.021	size_y = 40
		center_z = −4.598	size_z = 42
Oxygen-insensitive NAD(P)H nitroreductase	5J8G	center_x = 36.826	size_x = 23
		center_y = 45.915	size_y = 24
		center_z = −24.412	size_z = 22
Anthranilate--CoA ligase	5OE3	center_x = 38.132	size_x = 32
		center_y = −3.329	size_y = 26
		center_z = 14.677	size_z = 20

**Table 6:** Oregano essential oils’ physicochemical and organoleptic characteristics.

Plant Species	Properties			
	Yield (%)	Density	Color	Smell
*O. compactum*	2.40 ± 0.12	0.937	Light yellow	Strong aroma
*O. elongatum*	1.76 ± 0.09	0.893	Dark yellow	

**Table 7:** The chemical compositions of the EOs of the oreganos studied.

KI	Compounds	Area (%)	
		*O. compactum*	*O. elongatum*
930	Thujene <α->	1.01	0.17
939	Pinene <α->	0.99	1.52
954	Camphene	0.18	2.25
979	Pinene <β->	0.19	0.17
979	Octen-3-ol <1->	0.73	0.47
990	Myrcene	2.21	0.47
1002	Phellandrene <α->	0.4	0
1011	Carene <δ-3->	0.14	0
1017	Terpinene <α->	2.14	0.38
1024	Cymene <p->	15.84	42.56
1029	Phellandrene <β->	0.39	0
1029	Limonene	0.54	0.52
1059	Terpinene <γ->	22.89	2.98
1070	Sabinene hydrate <cis->	0.05	0.1
1072	Linalool oxide <cis->	0	0.21
1085	Cymenene <meta->	0.13	0
1086	Linalool oxide <trans->	0	0.56
1096	Linalool	2.66	3.42
1146	Camphor	0	0.23
1169	Borneol	0.24	8.58
1177	Terpinen-4-ol	0.37	0.57
1182	Cymen-8-ol <ρ->	0	0.28
1188	Terpineol <α->	0.63	0.17
1244	Carvacrol, methyl ether	0.15	0.1
1285	Bornyl acetate	0	0.14
1290	Thymol	10.21	28.43
1299	Carvacrol	30.89	0
1343	Piperitenone	0.45	0.17
1419	Caryophyllene <(E)->	3.63	1.67
1441	Aromadendrene	0	0.13
1454	Humulene <α->	0.23	0
1505	Bisabolene <β->	0.57	0
1513	Cadinene <γ->	0	0.16
1523	Cadinene <δ->	0.36	0.12
1578	Spathulenol	0	0.39
1583	Caryophyllene oxide	1.6	2.28
1686	Germacra-4(15),5,10(14)-trien-1-α-ol	0	0.25
1676	Cadalene	0	0.35
1733	Zerumbone	0	0.17
	Hydrocarbon monoterpenes	47.05	51.02
	Oxygenated monoterpenes	46.74	43.71
	Hydrocarbon sesquiterpenes	4.43	2.15
	Oxygenated sesquiterpenes	1.60	3.09
	Total	99.82	99.97

KI: Kovats retention index.

**Table 8:** MIC (µg/mL) of antibiotics evaluated by BD Phoenix and VITEX 2 for selected species.

Micro-Organisms	References	*MIC* (µg/mL) Identification and antibiogram instrument BD Phoenix™ VITEK 2											
		Gentamycin	Amoxicillin-Clavulanate	Vancomycin	Trimethoprim-Sulfamethoxazole	Penicillin G	Imipenem	Ampicillin	Ciprofloxacin	Ceftriaxone	Erythromycin	Tetracycline	Oxacillin
*S. epidermidis*	5994	2		>8	>4/76								
*S. aureus BLACT*	4IH2510	<0.5		2	<10								
*S. aureus 2220*	2DT2220	>8		>8	>4/76		>32				>8	>1	>4
*S. acidominimus*	7DT2108	≤250		<0.5		0.03							
*S. groupe D*	3EU9286	>1000		<0.5		0.13							
*S. agalactiae (B)*	7DT1887	≤250		>4		0.06							
*S. porcinus*	2EU9285	≤250		<0.5		0.06							
*E. faecalis*	2CQ9355	≤500		1	≤0.5/9.5								
*E. faecium*	13EU7181	≤500		>4	>4/76								
*A. baumannii*	7DT2404	≤1	≤2/2		≤1/19								
*A. baumannii 2410*	7DT2410	>8	>32/2		>8/152		>8	>16	>1	>4			
*E. coli*	3DT1938	2	8/2		≤1/19								
*E. coli ESBL*	2DT2057	2	>8/2		>4/76								
*E. coli ESBL 5765*	2DT5765	>16	>32/2		>320		1	>32	>4	>64			
*E. aerogenes*	07CQ164	≤1	8/2		≤1/19								
*E. cloacae*	02EV317	>4	>8/2		>4/76								
*E. cloacae 2280*	2DT2280	>16	>32/2		>8/152		>8	>32	>4	>64			
*C. koseri*	3DT2151	<1	>8/2		<20								
*K. pneumoniae*	3DT1823	≤1	≤2/2		≤1/19								
*K. pneumoniae 1015*	3DT1015	8	>32		40		8	>32	>4	>64			
*P. mirabilis*	2DS5461	2	≤2/2		>1/19								
*P. aeruginosa*	2DT2138	2	>8/2		4/76								
*P. aeruginosa 1124*	2DT1124	>4	>8/2		>4/76		>8	>32	>4	>64			
*P. fluorescence*	5442	4	>8/2		4/76								
*P. putida*	2DT2140	>4	>8/2		>4/76								
*S. marcescences*	375BR6	4	>8/2		>4/76								
*Salmonella* sp.	2CG5132	>4	8/2		>4/76								
*Shigella* sp.	7DS1513	>4	8/2		>4/76								
*Y. enterolitica*	ATCC27729	≤1	8/2		2/38								

**Table 9:** MIC (µg/mL) of Terbinafine.

Micro-Organisms	Terbinafine
*C. albicans*	12.500
*C. kyfer*	25.000
*C. krusei*	50.000
*C. parapsilosis*	6.250
*C. tropicalis*	12.500
*C. dubliniensis*	3.125
*S. cerevisiae*	3.125
*A. niger*	3.125

**Table 10:** MIC and MBC (µg/mL) for EO of oregano studied.

Micro-Organisms		*O. elongatum*		*O. compactum*	
		*MIC*	*MBC*	*MIC*	*MBC*
*Gram-Positive Cocci*	*S. epidermidis*	2500	2500	2500	5000
	*S. aureus BLACT*	2500	2500	1200	1200
	*S. aureus 2220*	>5000	>5000	5000	5000
	*S. acidominimus*	300	600	75	150
	*S. groupe D*	600	600	600	600
	*S. agalactiae (B)*	1200	2500	600	1200
	*S. porcinus*	2500	2500	5000	5000
	*E. faecalis*	600	1200	300	300
	*E. faecium*	5000	5000	5000	5000
*Gram-Negative Bacilli*	*A. baumannii*	300	300	150	300
	*A. baumannii 2410*	300	300	300	300
	*E. coli*	1200	1200	300	600
	*E. coli ESBL*	1200	1200	600	600
	*E. coli ESBL 5765*	1200	1200	600	600
	*E. aerogenes*	1200	2500	1200	1200
	*E. cloacae*	75	150	150	300
	*E. cloacae 2280*	150	300	150	300
	*C. koseri*	75	150	150	150
	*K. pneumoniae*	600	1200	600	600
	*K. pneumoniae 1015*	600	1200	600	600
	*P. mirabilis*	2500	5000	1200	2500
	*P. aeruginosa*	300	300	75	150
	*P. aeruginosa 1124*	300	300	150	150
	*P. fluorescence*	1200	2500	600	600
	*P. putida*	2500	5000	600	1200
	*S. marcescences*	1200	2500	2500	5000
	*Salmonella* sp.	1200	2500	150	300
	*Shigella* sp	600	1200	300	600
	*Y. enterolitica*	300	300	300	600

**Table 11:** MIC and MFC (µg/mL) of EOs of oregano studied.

Micro-Organisms	*O. elongatum*		*O. compactum*	
	*MIC*	*MFC*	*MIC*	*MFC*
*C. albicans*	600	600	600	600
*C. kyfer*	2500	2500	1200	1200
*C. krusei*	5000	5000	2500	5000
*C. parapsilosis*	1200	1200	600	1200
*C. tropicalis*	1200	1200	600	600
*C. dubliniensis*	1200	1200	150	300
*S. cerevisiae*	2500	2500	2500	2500
*A. niger*	150	300	75	150

**Table 12:** FICI values of EO combinations studied with antibiotics.

Strains	Compounds		MIC (µg/mL)				FIC		FICI	Output
			Alone		Combinations					
	EOs	ATBs *	EOs	ATBs	EOs	ATBs	EOs	ATBs		
*K. pneumoniae 5694*	*O. compactum*	GEN	0.6	128	0.6	4	1.00	0.031	1.031	Indifference
		IMP	0.6	16	1.2	4	2.00	0.250	2.250	Indifference
		AMP	0.6	>2048	-	-	-	-	-	-
		CIP	0.6	1024	0.6	4	1.00	0.004	1.004	Indifference
		CRO	0.6	1024	1.2	4	2.00	0.004	2.004	Indifference
		ERY	0.6	128	0.3	4	0.50	0.031	0.531	Additive
		TET	0.6	4	1.2	4	2.00	1.000	3.000	Indifference
		AMX	0.6	128	0.15	4	0.25	0.031	0.281	Synergistic
		VAN	0.6	128	0.6	128	1.00	1.000	2.000	Indifference
	*O. elongatum*	GEN	0.6	128	0.075	4	0.13	0.031	0.156	Synergistic
		IMP	0.6	16	0.3	8	0.50	0.500	1.000	Additive
		AMP	0.6	>2048	-	-	-	-	-	-
		CIP	0.6	1024	0.075	4	0.13	0.004	0.129	Synergistic
		CRO	0.6	1024	1.2	4	2.00	0.004	2.004	Indifference
		ERY	0.6	128	0.075	4	0.13	0.031	0.156	Synergistic
		TET	0.6	4	2.5	4	4.17	1.000	5.167	antagonism
		AMX	0.6	128	0.3	4	0.50	0.031	0.531	Additive
		VAN	0.6	128	0.15	128	0.25	1.000	1.250	Indifference
*P. aerogenosa 5824*	*O. compactum*	GEN	0.6	16	0.6	4	1.00	0.250	1.250	Indifference
		IMP	0.6	32	0.075	4	0.13	0.125	0.250	Synergistic
		AMP	0.6	1024	0.075	64	0.13	0.063	0.188	Synergistic
		CIP	0.6	256	0.3	16	0.50	0.063	0.563	Additive
		CRO	0.6	64	0.075	4	0.13	0.063	0.188	Synergistic
		ERY	0.6	256	0.075	4	0.13	0.016	0.141	Synergistic
		TET	0.6	128	0.6	16	1.00	0.125	1.125	Indifference
		AMX	0.6	2048	2.5	2048	4.17	1.000	5.167	antagonism
		VAN	0.6	256	0.6	256	1.00	1.000	2.000	Indifference
	*O. elongatum*	GEN	1.2	8	0.6	4	0.50	0.500	1.000	Additive
		IMP	1.2	32	0.3	4	0.25	0.125	0.375	Synergistic
		AMP	1.2	1024	0.075	4	0.06	0.004	0.066	Synergistic
		CIP	1.2	256	0.15	32	0.13	0.125	0.250	Synergistic
		CRO	1.2	64	0.075	4	0.06	0.063	0.125	Synergistic
		ERY	1.2	256	0.075	4	0.06	0.016	0.078	Synergistic
		TET	1.2	128	0.075	4	0.06	0.031	0.094	Synergistic
		AMX	1.2	2048	2.5	2048	2.08	1.000	3.083	Indifference
		VAN	1.2	256	0.075	64	0.06	0.250	0.313	Synergistic
*E. coli ESBL 5765*	*O. compactum*	GEN	0.6	64	0.6	64	1.00	1.000	2.000	Indifference
		IMP	0.6	4	0.15	4	0.25	1.000	1.250	Indifference
		AMP	0.6	>2048	-	-	-	-	-	-
		CIP	0.6	64	0.6	16	1.00	0.250	1.250	Indifference
		CRO	0.6	16	0.075	4	0.13	0.250	0.375	Synergistic
		ERY	0.6	>2048	-	-	-	-	-	-
		TET	0.6	8	0.15	4	0.25	0.500	0.750	Additive
		AMX	0.6	>2048	-	-	-	-	-	-
		VAN	0.6	512	1.2	256	2.00	0.500	2.500	Indifference
	*O. elongatum*	GEN	1.2	64	0.6	64	0.50	1.000	1.500	Indifference
		IMP	1.2	4	0.6	4	0.50	1.000	1.500	Indifference
		AMP	1.2	>2048	-	-	-	-	-	-
		CIP	1.2	64	0.6	16	0.50	0.250	0.750	Additive
		CRO	1.2	16	0.3	8	0.25	0.500	0.750	Additive
		ERY	1.2	>2048	-	-	-	-	-	-
		TET	1.2	8	2.5	4	2.08	0.500	2.583	Indifference
		AMX	1.2	>2048	-	-	-	-	-	-
		VAN	1.2	512	0.15	256	0.13	0.500	0.625	Additive
*A. baumannii 2410*	*O. compactum*	GEN	0.3	1024	0.3	1024	1.00	1.000	2.000	Indifference
		IMP	0.3	512	0.15	256	0.50	0.500	1.000	Additive
		AMP	0.3	2048	0.6	256	2.00	0.125	2.125	Indifference
		CIP	0.3	256	0.6	16	2.00	0.063	2.063	Indifference
		CRO	0.3	512	0.6	512	2.00	1.000	3.000	Indifference
		ERY	0.3	128	0.15	4	0.50	0.031	0.531	Additive
		TET	0.3	16	0.6	4	2.00	0.250	2.250	Indifference
		AMX	0.3	512	0.15	128	0.50	0.250	0.750	Additive
		VAN	0.3	256	0.3	128	1.00	0.500	1.500	Indifference
	*O. elongatum*	GEN	0.6	1024	0.6	1024	1.00	1.000	2.000	Indifference
		IMP	0.6	512	0.3	256	0.50	0.500	1.000	Additive
		AMP	0.6	2048	0.3	256	0.50	0.125	0.625	Additive
		CIP	0.6	256	0.6	128	1.00	0.500	1.500	Indifference
		CRO	0.6	512	0.6	512	1.00	1.000	2.000	Indifference
		ERY	0.6	128	0.15	4	0.25	0.031	0.281	Synergistic
		TET	0.6	16	0.6	4	1.00	0.250	1.250	Indifference
		AMX	0.6	512	0.15	64	0.25	0.125	0.375	Synergistic
		VAN	0.6	256	0.3	128	0.50	0.500	1.000	Additive
*S. aureus 2220*	*O. compactum*	GEN	1.2	16	0.3	4	0.25	0.250	0.500	Synergistic
		IMP	1.2	64	0.15	4	0.13	0.063	0.188	Synergistic
		AMP	1.2	16	0.6	4	0.50	0.250	0.750	Additive
		CIP	1.2	32	0.6	4	0.50	0.125	0.625	Additive
		CRO	1.2	16	0.075	4	0.06	0.250	0.313	Synergistic
		ERY	1.2	16	0.6	4	0.50	0.250	0.750	Additive
		TET	1.2	16	0.075	4	0.06	0.250	0.313	Synergistic
		AMX	1.2	64	0.075	4	0.06	0.063	0.125	Synergistic
		VAN	1.2	64	0.075	4	0.06	0.063	0.125	Synergistic
	*O. elongatum*	GEN	2.5	16	0.6	4	0.24	0.250	0.490	Synergistic
		IMP	2.5	64	0.3	4	0.12	0.063	0.183	Synergistic
		AMP	2.5	16	0.6	4	0.24	0.250	0.490	Synergistic
		CIP	2.5	32	0.15	4	0.06	0.125	0.185	Synergistic
		CRO	2.5	16	0.6	4	0.24	0.250	0.490	Synergistic
		ERY	2.5	16	1.2	4	0.48	0.250	0.730	Additive
		TET	2.5	16	0.6	4	0.24	0.250	0.490	Synergistic
		AMX	2.5	64	0.15	4	0.06	0.063	0.123	Synergistic
		VAN	2.5	64	0.075	4	0.03	0.063	0.093	Synergistic
*E. cloacae 2280*	*O. compactum*	GEN	0.6	32	0.3	4	0.50	0.125	0.625	Additive
		IMP	0.6	128	1.2	4	2.00	0.031	2.031	Indifference
		AMP	0.6	2048	0.3	512	0.50	0.250	0.750	Additive
		CIP	0.6	1024	0.3	256	0.50	0.250	0.750	Additive
		CRO	0.6	512	0.6	4	1.00	0.008	1.008	Indifference
		ERY	0.6	16	0.3	4	0.50	0.250	0.750	Additive
		TET	0.6	1024	0.15	8	0.25	0.008	0.258	Synergistic
		AMX	0.6	32	1.2	4	2.00	0.125	2.125	Indifference
		VAN	0.6	1024	0.3	512	0.50	0.500	1.000	Additive
	*O. elongatum*	GEN	0.3	32	0.075	4	0.25	0.125	0.375	Synergistic
		IMP	0.3	128	0.075	4	0.25	0.031	0.281	Synergistic
		AMP	0.3	2048	0.075	512	0.25	0.250	0.500	Synergistic
		CIP	0.3	1024	0.075	128	0.25	0.125	0.375	Synergistic
		CRO	0.3	512	0.6	512	2.00	1.000	3.000	Indifference
		ERY	0.3	16	0.6	4	2.00	0.250	2.250	Indifference
		TET	0.3	1024	0.15	256	0.50	0.250	0.750	Additive
		AMX	0.3	32	0.6	4	2.00	0.125	2.125	Indifference
		VAN	0.3	1024	0.3	256	1.00	0.250	1.250	Indifference

* Antibiotics (ATBs): Gentamicin (GEN); Imipenem (IMP); Ampicillin (AMP); Ciprofloxacin (CIP); Ceftriaxone (CRO); Erythromycin (ERY); Tetracycline (TET); Amoxicillin (AMX); Vancomycin (VAN).

**Table 13:** In silico PASS and ADME analysis of oregano EOs studied.

	Absorption					Distribution				Metabolism							Excretion	
Compounds	Water Solubility	Caco2 Permeability	Intestinal Absorption (Human)	Skin Permeability	P-glycoprotein Substrate/ P-glycoprotein I and II Inhibitor	VDss (Human)	Fraction Unbound (Human)	BBB Permeability	CNS Permeability	CYP	Inhibitor						Total Clearance	Renal OCT2 Substrate
										Substrate		1A2	2C19	2C9	2D6	3A4		
										2D6	3A4							
Borneol	−2.462	1.484	93.439	−2.174	No	0.337	0.486	0.646	−2.331	No		No	No				1.035	No
Carvacrol	−2.789	1.606	90.843	−1.62		0.512	0.203	0.407	−1.664			Yes					0.207	No
(E)-caryophyllene	−5.555	1.423	94.845	−1.58		0.652	0.263	0.733	−2.172			No					1.088	No
ɣ-terpinene	−3.941	1.414	96.219	−1.489		0.412	0.42	0.754	−2.049								0.217	No
Linalool	−2.612	1.493	93.163	−1.737		0.152	0.484	0.598	−2.339								0.446	No
p-cymene	−4.081	1.527	93.544	−1.192		0.697	0.159	0.478	−1.397			Yes					0.239	No
Thymol	−2.789	1.606	90.843	−1.62		0.512	0.203	0.407	−1.664			Yes					0.211	No

**Table 14:** In silico analysis of the predictive toxicity (ProTox II) of the oregano EOs studied.

Compounds	AMES Toxicity	Max. Tolerated Dose (Human)	hERG I Inhibitor and hERG II Inhibitor	Oral Rat Acute Toxicity (LD50)	Oral Rat Chronic Toxicity	Hepatotoxicity	Skin Sensitization	*T. Pyriformis* Toxicity	Minnow Toxicity
Borneol	No	0.577	No	1.707	1.877	No	Yes	0.175	1.727
Carvacrol		1.007		2.074	2.212	Yes	Yes	0.387	1.213
(E)-caryophyllene		0.351		1.617	1.416	No	Yes	1.401	0.504
ɣ-terpinene		0.756		1.766	2.394	No	No	0.627	0.906
Linalool		0.774		1.704	2.024	No	Yes	0.515	1.277
p-cymene		0.903		1.827	2.328	No	Yes	0.462	0.869
Thymol		1.007		2.074	2.212	Yes	Yes	0.387	1.213

**Table 15:** Details of binding affinities of antimicrobial target proteins to selected ligands.

Ligands\Targets		1JZQ	2VEG	2ZDQ	3RAE	3SRW	3UDI	1KZN	5j8g	5oe3	4URN
Compounds	Borneol	−5.1	−4.4	−5.7	−4.6	−5.5	−4.9	−4.4	−4.1	−4.7	−4.5
	Carvacrol	−5.8	−4.9	−8.2	−5	−5.8	−5.1	−6	−4.3	−6.2	−5.6
	(E)-Caryophyllene	−6.8	−5.4	−6.5	−6	−8.1	−6	−6.3	−4.5	−6	−6.5
	ɣ-terpinene	−5.3	−4.7	−7.7	−4.4	−5.7	−4.7	−5.8	−4.3	−5.9	−5.1
	Linalool	−5.3	−4.4	−6.2	−4.7	−5.7	−4.6	−5.4	−4.1	−4.8	−4.9
	p-cymene	−5.5	−4.5	−8	−4.4	−5.6	−4.7	−5.8	−4.3	−6.1	−5.1
	Thymol	−5.4	−4.8	−7.7	−4.8	−5.7	−5.3	−6.3	−4.1	−6.4	−5.2
Antibiotics	Amoxicillin	−7.9	−6.4	−8.8	−6.7	−8.9	−8.5	−7.2	−6.1	−8.2	−6.7
	Ampicillin	−7.8	−6	−8.3	−6.6	−8.7	8.3	−7.1	−5.6	−7.8	−7.6
	Ceftriaxone	−8.4	−6.6	−6.9	−6.8	−8	−8	−7	−6	−7.7	7.5
	Ciprofloxacin	−8.1	−6.6	−7.5	−6.6	−9.4	−8	−7.2	−6.1	−6.7	−7.8
	Erythromycin	−9.1	−5.7	−4.6	−6.8	−5.7	−6.2	−5.8	−5.6	−6.1	−5.6
	Gentamicin	−8	−5.8	−5.6	−5.9	−7.7	−7	−6.4	−5.2	−5.9	−6.3
	Imipinem	−6.8	−6	−6.9	−5.8	−7.1	−6.9	−5.8	−5.2	−6.3	−6.5
	Tetracycline	−9.2	−7.1	−7	−7.1	−7.2	−9.6	−8.3	−6.2	−7.8	−7.8
	Vancomycin				−6.7	−6.4		−6.1		−5.2	−6.4

## Data Availability

Data are contained within this article.

## References

[1] Podolsky S.H. (2015). The Antibiotic Era: Reform, Resistance, and the Pursuit of a Rational Therapeutics.

[2] Fatima Z., Purkait D., Rehman S., Rai S., Hameed S. (2023). Multidrug Resistance: A Threat to Antibiotic Era. Biological and Environmental Hazards, Risks, and Disasters.

[3] Browne A.J., Chipeta M.G., Haines-Woodhouse G., Kumaran E.P.A., Hamadani B.H.K., Zaraa S., Henry N.J., Deshpande A., Reiner R.C., Day N.P.J. (2021). Global Antibiotic Consumption and Usage in Humans, 2000–18: A Spatial Modelling Study. Lancet Planet. Health.

[4] Bouki C., Venieri D., Diamadopoulos E. (2013). Detection and Fate of Antibiotic Resistant Bacteria in Wastewater Treatment Plants: A Review. Ecotoxicol. Environ. Saf..

[5] Merrikh H., Kohli R.M. (2020). Targeting Evolution to Inhibit Antibiotic Resistance. FEBS J..

[6] Medina M., Legido-Quigley H., Hsu L.Y., Masys A.J., Izurieta R., Reina Ortiz M. (2020). Antimicrobial Resistance in One Health. Global Health Security.

[7] Elmaidomy A.H., Shady N.H., Abdeljawad K.M., Elzamkan M.B., Helmy H.H., Tarshan E.A., Adly A.N., Hussien Y.H., Sayed N.G., Zayed A. (2022). Antimicrobial Potentials of Natural Products against Multidrug Resistance Pathogens: A Comprehensive Review. RSC Adv..

[8] Pino-Otín M.R., Gan C., Terrado E., Sanz M.A., Ballestero D., Langa E. (2022). Antibiotic Properties of Satureja Montana L. Hydrolate in Bacteria and Fungus of Clinical Interest and Its Impact in Non-Target Environmental Microorganisms. Sci. Rep..

[9] H Moreno P.R., da Costa-Issa F., Rajca-Ferreira A.K., Pereira M.A.A., Kaneko T.M. (2013). Native Brazilian Plants Against Nosocomial Infections: A Critical Review on Their Potential and the Antimicrobial Methodology. Curr. Top. Med. Chem..

[10] Cheesman M.J., Ilanko A., Blonk B., Cock I.E. (2017). Developing New Antimicrobial Therapies: Are Synergistic Combinations of Plant Extracts/Compounds with Conventional Antibiotics the Solution?. Pharmacogn. Rev..

[11] Chung P.Y., Navaratnam P., Chung L.Y. (2011). Synergistic Antimicrobial Activity between Pentacyclic Triterpenoids and Antibiotics against Staphylococcus Aureus Strains. Ann. Clin. Microbiol. Antimicrob..

[12] Rahman A.U., Choudhary M.I. (2018). Frontiers in Anti-Infective Drug Discovery.

[13] Iseppi R., Mariani M., Condò C., Sabia C., Messi P. (2021). Essential Oils: A Natural Weapon against Antibiotic-Resistant Bacteria Responsible for Nosocomial Infections. Antibiotics.

[14] Langeveld W.T., Veldhuizen E.J.A., Burt S.A. (2014). Synergy between Essential Oil Components and Antibiotics: A Review. Crit. Rev. Microbiol..

[15] Tyers M., Wright G.D. (2019). Drug Combinations: A Strategy to Extend the Life of Antibiotics in the 21st Century. Nat. Rev. Microbiol..

[16] Prakash B., Kumar A., Singh P.P., Songachan L.S., Prakash B. (2020). Antimicrobial and Antioxidant Properties of Phytochemicals: Current Status and Future Perspective. Functional and Preservative Properties of Phytochemicals.

[17] Uddin T.M., Chakraborty A.J., Khusro A., Zidan B.R.M., Mitra S., Emran T.B., Dhama K., Ripon M.K.H., Gajdács M., Sahibzada M.U.K. (2021). Antibiotic Resistance in Microbes: History, Mechanisms, Therapeutic Strategies and Future Prospects. J. Infect. Public Health.

[18] Sharifi-Rad M., Berkay Yılmaz Y., Antika G., Salehi B., Tumer T.B., Kulandaisamy Venil C., Das G., Patra J.K., Karazhan N., Akram M. (2021). Phytochemical Constituents, Biological Activities, and Health-Promoting Effects of the Genus Origanum. Phytother. Res. PTR.

[19] Jedidi S., Sebai H., Jedidi S., Sebai H. (2024). Phytochemistry, Medicinal Uses, and Beneficial Nutritional Effects of Essential Oils.

[20] Çalışkan M.M. (2023). Ufuk Koca *Origanum* sp. Medicinal Plants of Turkey.

[21] Jaiswal A., Verma M., Pandey S., Chitnavis S., Pathak R., Shah K., Chauhan D.N., Chauhan N.S. (2024). Health Benefits of Oregano Extract. Plant-Based Bioactive Compounds and Food Ingredients.

[22] Padulosi S. (1997). Oregano. Promoting the Conservation and Use of Underutilized and Neglected Crops.

[23] Bellakhdar J. (1997). La Pharmacopée Traditionnelle Marocaine.

[24] Ouedrhiri W., Balouiri M., Bouhdid S., Moja S., Chahdi F.O., Taleb M., Greche H. (2016). Mixture Design of Origanum Compactum, Origanum Majorana and Thymus Serpyllum Essential Oils: Optimization of Their Antibacterial Effect. Ind. Crops Prod..

[25] Bouyahya A., Dakka N., Talbaoui A., Moussaoui N.E., Abrini J., Bakri Y. (2018). Phenolic Contents and Antiradical Capacity of Vegetable Oil from *Pistacia lentiscus* (L). J. Mater. Environ. Sci..

[26] Laghmouchi Y., Belmehdi O., Senhaji N.S., Abrini J. (2018). Chemical Composition and Antibacterial Activity of Origanum Compactum Benth. Essential Oils from Different Areas at Northern Morocco. S. Afr. J. Bot..

[27] Wilson J. (2019). Infection Control in Clinical Practice Updated Edition.

[28] Kovats E.S. (1965). Gas Chromatographic Characterization of Organic Substances in the Retention Index System. Adv. Chromatogr..

[29] Adams R.P. (2007). Identification of Essential Oil Components by Gas Chromatography/Mass Spectrometry.

[30] Hübschmann H.-J. (2015). Handbook of GC-MS: Fundamentals and Applications.

[31] Balouiri M., Sadiki M., Ibnsouda S.K. (2016). Methods for in Vitro Evaluating Antimicrobial Activity: A Review. J. Pharm. Anal..

[32] Bissel S.J., Winkler C.C., DelTondo J., Wang G., Williams K., Wiley C.A. (2014). Coxsackievirus B4 Myocarditis and Meningoencephalitis in Newborn Twins. Neuropathology.

[33] Dinarvand M., Spain M.P., Vafaee F. (2020). Pharmacodynamic Functions of Synthetic Derivatives for Treatment of Methicillin-Resistant Staphylococcus Aureus (MRSA) and Mycobacterium Tuberculosis. Front. Microbiol..

[34] Armengol E., Kragh K.N., Tolker-Nielsen T., Sierra J.M., Higazy D., Ciofu O., Viñas M., Høiby N. (2023). Colistin Enhances Rifampicin’s Antimicrobial Action in Colistin-Resistant Pseudomonas Aeruginosa Biofilms. Antimicrob. Agents Chemother..

[35] Li Z., Wan H., Shi Y., Ouyang P. (2004). Personal Experience with Four Kinds of Chemical Structure Drawing Software: Review on ChemDraw, ChemWindow, ISIS/Draw, and ChemSketch. J. Chem. Inf. Comput. Sci..

[36] Filimonov D.A., Lagunin A.A., Gloriozova T.A., Rudik A.V., Druzhilovskii D.S., Pogodin P.V., Poroikov V.V. (2014). Prediction of the Biological Activity Spectra of Organic Compounds Using the Pass Online Web Resource. Chem. Heterocycl. Compd..

[37] Daina A., Michielin O., Zoete V. (2017). SwissADME: A Free Web Tool to Evaluate Pharmacokinetics, Drug-Likeness and Medicinal Chemistry Friendliness of Small Molecules. Sci. Rep..

[38] Alam A., Jawaid T., Alam P. (2021). In Vitro Antioxidant and Anti-Inflammatory Activities of Green Cardamom Essential Oil and in Silico Molecular Docking of Its Major Bioactives. J. Taibah Univ. Sci..

[39] Banerjee P., Eckert A.O., Schrey A.K., Preissner R. (2018). ProTox-II: A Webserver for the Prediction of Toxicity of Chemicals. Nucleic Acids Res..

[40] Van Der Spoel D., Lindahl E., Hess B., Groenhof G., Mark A.E., Berendsen H.J.C. (2005). GROMACS: Fast, Flexible, and Free. J. Comput. Chem..

[41] Salam M.A., Al-Amin M.Y., Salam M.T., Pawar J.S., Akhter N., Rabaan A.A., Alqumber M.A.A. (2023). Antimicrobial Resistance: A Growing Serious Threat for Global Public Health. Healthcare.

[42] Mancuso G., Midiri A., Gerace E., Biondo C. (2021). Bacterial Antibiotic Resistance: The Most Critical Pathogens. Pathogens.

[43] Chang Q., Wang W., Regev-Yochay G., Lipsitch M., Hanage W.P. (2015). Antibiotics in Agriculture and the Risk to Human Health: How Worried Should We Be?. Evol. Appl..

[44] El Atki Y., Aouam I., El Kamari F., Taroq A., Lyoussi B., Oumokhtar B., Abdellaoui A. (2020). Phytochemistry, Antioxidant and Antibacterial Activities of Two Moroccan Teucrium Polium L. Subspecies: Preventive Approach against Nosocomial Infections. Arab. J. Chem..

[45] Tagnaout I., Zerkani H., Hadi N., El Moumen B., El Makhoukhi F., Bouhrim M., Al-Salahi R., Nasr F.A., Mechchate H., Zair T. (2022). Chemical Composition, Antioxidant and Antibacterial Activities of Thymus Broussonetii Boiss and *Thymus capitatus* (L.) Hoffmann and Link Essential Oils. Plants.

[46] Basavegowda N., Baek K.-H. (2022). Combination Strategies of Different Antimicrobials: An Efficient and Alternative Tool for Pathogen Inactivation. Biomedicines.

[47] Ez-Zriouli R. (2021). Assessment of Bioactive Compounds, Antibacterial Potential and Acute Toxicity of a Volatile Origanum Compactum Extract, an Endemic Plant of Northern Morocco. Arab. J. Med. Aromat. Plants.

[48] Yousif L., Belmehdi O., Abdelhakim B., Skali Senhaji N., Abrini J. (2021). Does the Domestication of *Origanum compactum* (Benth) Affect Its Chemical Composition and Antibacterial Activity?. Flavour Fragr. J..

[49] Hafssa H., Abderrahmane R., Nabila S., Guido F., Roberta A., Hichem B. (2021). Comparative Study of the Variability of Chemical Composition and Antibacterial Activity between Two Moroccan Endemic Species Essential Oils: Origanum grosiiPau & Font Quer and *Origanum elongatum* (Bonnet) Emberger & Maire. Egypt. J. Chem..

[50] El Kharraf S., El-Guendouz S., Farah A., Bennani B., Mateus M.C., El Hadrami E.M., Miguel M.G. (2021). Hydrodistillation and Simultaneous Hydrodistillation-Steam Distillation of Rosmarinus Officinalis and Origanum Compactum: Antioxidant, Anti-Inflammatory, and Antibacterial Effect of the Essential Oils. Ind. Crops Prod..

[51] Wogiatzi E., Gougoulias N., Papachatzis A., Vagelas I., Chouliaras N. (2009). Chemical Composition and Antimicrobial Effects of Greek Origanum Species Essential Oil. Biotechnol. Biotechnol. Equip..

[52] Figuérédo G., Cabassu P., Chalchat J.-C., Pasquier B. (2006). Studies of Mediterranean Oregano Populations. VI: Chemical Composition of Essential Oils of Origanum Elongatum Emberger et Maire from Morocco. J. Essent. Oil Res..

[53] Bakha M., El Mtili N., Machon N., Aboukhalid K., Amchra F.Z., Khiraoui A., Gibernau M., Tomi F., Al Faiz C. (2018). Intraspecific Chemical Variability of the Essential Oils of Moroccan Endemic *Origanum elongatum* L. (Lamiaceae) from Its Whole Natural Habitats. Arab. J. Chem..

[54] Oualili H., Nmila R., Chibi M., Mricha A., Rchid H., Rchid H. (2019). Chemical Composition and Antioxidant Activity of Origanum Elongatum Essential Oil. Pharmacogn. Res..

[55] Prestinaci F., Pezzotti P., Pantosti A. (2015). Antimicrobial Resistance: A Global Multifaceted Phenomenon. Pathog. Glob. Health.

[56] Rao J., Chen B., McClements D.J. (2019). Improving the Efficacy of Essential Oils as Antimicrobials in Foods: Mechanisms of Action. Annu. Rev. Food Sci. Technol..

[57] Bagamboula C.F., Uyttendaele M., Debevere J. (2004). Inhibitory Effect of Thyme and Basil Essential Oils, Carvacrol, Thymol, Estragol, Linalool and p-Cymene towards Shigella Sonnei and S. Flexneri. Food Microbiol..

[58] Ejaz A., Waliat S., Arshad M.S., Khalid W., Khalid M.Z., Rasul Suleria H.A., Luca M.-I., Mironeasa C., Batariuc A., Ungureanu-Iuga M. (2023). A Comprehensive Review of Summer Savory (*Satureja hortensis* L.): Promising Ingredient for Production of Functional Foods. Front. Pharmacol..

[59] Rosato A., Piarulli M., Corbo F., Muraglia M., Carone A., Vitali M.E., Vitali C. (2010). In Vitro Synergistic Action of Certain Combinations of Gentamicin and Essential Oils. Curr. Med. Chem..

[60] Scandorieiro S., de Camargo L.C., Lancheros C.A.C., Yamada-Ogatta S.F., Nakamura C.V., de Oliveira A.G., Andrade C.G.T.J., Duran N., Nakazato G., Kobayashi R.K.T. (2016). Synergistic and Additive Effect of Oregano Essential Oil and Biological Silver Nanoparticles against Multidrug-Resistant Bacterial Strains. Front. Microbiol..

[61] Oumam N., Rais A., Benaamara A., Mohamed Abdoul-Latif F., Ayoub A., André P., Tarik A., Abourriche A. (2021). Chemical Composition and Biological Activities of Essential Oils and Solvent Extracts of *Origanum elongatum* from Morocco. Pharmacologyonline.

[62] Lambert R.J.W., Skandamis P.N., Coote P.J., Nychas G.-J. (2001). A Study of the Minimum Inhibitory Concentration and Mode of Action of Oregano Essential Oil, Thymol and Carvacrol. J. Appl. Microbiol..

[63] Amakran A., Hamoudane M., Ramdan B., Lamarti A., Pagniez F., Le Pape P., Nhiri M. (2014). Antifungal Activity of the Essential Oil of Origanum Elongatum on Candida, Aspergillus and Rhizopus Species. J. Mycol. Médicale J. Med. Mycol..

[64] Hyldgaard M., Mygind T., Meyer R. (2012). Essential Oils in Food Preservation: Mode of Action, Synergies, and Interactions with Food Matrix Components. Front. Microbiol..

[65] Becerril R., Nerín C., Gómez-Lus R. (2012). Evaluation of Bacterial Resistance to Essential Oils and Antibiotics After Exposure to Oregano and Cinnamon Essential Oils. Foodborne Pathog. Dis..

[66] Ju J., Xie Y., Yu H., Guo Y., Cheng Y., Qian H., Yao W. (2022). Synergistic Interactions of Plant Essential Oils with Antimicrobial Agents: A New Antimicrobial Therapy. Crit. Rev. Food Sci. Nutr..

[67] Silva S.L., Araújo F.S.M., Silva P.O.A., Silva E.V.A., Bezerra M.M.S.L., Diniz A.F., Oliveira D.M., Jesus H.O., Nascimento Junior B.B., Medeiros L.A.D.M. (2023). Evaluation of the Antimicrobial Effect of the Origanum Vulgare L Essential Oil on Strains of Klebsiella Pneumoniae. Braz. J. Biol..

[68] Maggini V., Pesavento G., Maida I., Nostro A.L., Calonico C., Sassoli C., Perrin E., Fondi M., Mengoni A., Chiellini C. (2017). Exploring the Effect of the Composition of Three Different Oregano Essential Oils on the Growth of Multidrug-Resistant Cystic Fibrosis Pseudomonas Aeruginosa Strains. Nat. Prod. Commun..

[69] Aelenei P., Miron A., Trifan A., Bujor A., Gille E., Aprotosoaie A.C. (2016). Essential Oils and Their Components as Modulators of Antibiotic Activity against Gram-Negative Bacteria. Medicines.

[70] Fayed B., Jagal J., Cagliani R., Kedia R.A., Elsherbeny A., Bayraktutan H., Khoder G., Haider M. (2023). Co-Administration of Amoxicillin-Loaded Chitosan Nanoparticles and Inulin: A Novel Strategy for Mitigating Antibiotic Resistance and Preserving Microbiota Balance in Helicobacter Pylori Treatment. Int. J. Biol. Macromol..

[71] Ojkic N., Lilja E., Direito S., Dawson A., Allen R.J., Waclaw B. (2020). A Roadblock-and-Kill Mechanism of Action Model for the DNA-Targeting Antibiotic Ciprofloxacin. Antimicrob. Agents Chemother..

[72] Mitscher L.A. (2005). Bacterial Topoisomerase Inhibitors:  Quinolone and Pyridone Antibacterial Agents. Chem. Rev..

[73] Alshammari F., Alshammari B., Moin A., Alamri A., Al Hagbani T., Alobaida A., Baker A., Khan S., Rizvi S.M.D. (2021). Ceftriaxone Mediated Synthesized Gold Nanoparticles: A Nano-Therapeutic Tool to Target Bacterial Resistance. Pharmaceutics.

[74] Furr M., Reed S. (2015). Equine Neurology.

[75] Dowling A., O’dwyer J., Adley C. (2017). Antibiotics: Mode of Action and Mechanisms of Resistance. Antimicrob. Res. Nov. Bioknowledge Educ. Programs.

[76] Khan S., Nazir M., Raiz N., Saleem M., Zengin G., Fazal G., Saleem H., Mukhtar M., Tousif M.I., Tareen R.B. (2019). Phytochemical Profiling, in Vitro Biological Properties and in Silico Studies on *Caragana ambigua* Stocks (Fabaceae): A Comprehensive Approach. Ind. Crops Prod..

[77] Duffy F.J., Devocelle M., Shields D.C. (2015). Computational Approaches to Developing Short Cyclic Peptide Modulators of Protein–Protein Interactions. Meth. Mol. Biol..

[78] Daina A., Michielin O., Zoete V. (2014). iLOGP: A Simple, Robust, and Efficient Description of n-Octanol/Water Partition Coefficient for Drug Design Using the GB/SA Approach. J. Chem. Inf. Model..

[79] Lipinski C.A., Lombardo F., Dominy B.W., Feeney P.J. (2001). Experimental and Computational Approaches to Estimate Solubility and Permeability in Drug Discovery and Development Settings1. Adv. Drug Deliv. Rev..

[80] Xiao G., Li J., Sun Z. (2023). The Combination of Antibiotic and Non-Antibiotic Compounds Improves Antibiotic Efficacy against Multidrug-Resistant Bacteria. Int. J. Mol. Sci..

[81] Khameneh B., Iranshahy M., Soheili V., Fazly Bazzaz B.S. (2019). Review on Plant Antimicrobials: A Mechanistic Viewpoint. Antimicrob. Resist. Infect. Control.

[82] Zheng D., Huang C., Huang H., Zhao Y., Khan M.R.U., Zhao H., Huang L. (2020). Antibacterial Mechanism of Curcumin: A Review. Chem. Biodivers..

[83] Baran A., Kwiatkowska A., Potocki L. (2023). Antibiotics and Bacterial Resistance—A Short Story of an Endless Arms Race. Int. J. Mol. Sci..

